# An Attack and Defense Strategy for Osteoarthritis Repair: Constructing a Trace Element Modulated Hydrogel to Mitigate Ferroptosis and Promote Cartilage Matrix Reconstruction

**DOI:** 10.34133/research.0979

**Published:** 2025-11-11

**Authors:** Wenhui Hu, Fei Kang, Yuheng Li, Yixiang Xu, Xiaoming Li, Jing Zhang, Jie Liao, Jingjin Dai, Xiaoshan Gong, Jianmei Li, Xuan Yao, Shiwu Dong

**Affiliations:** ^1^ Department of Biomedical Materials Science, College of Biomedical Engineering, Third Military Medical University, Chongqing 400038, China.; ^2^ State Key Laboratory of Trauma and Chemical Poisoning, Third Military Medical University, Chongqing 400038, China.; ^3^ Department of Basic Medicine, Frontier Medical Service Training Brigade, Third Military Medical University, Changji, Xinjiang 831200, China.; ^4^ Department of Military Traffic Medicine, Daping Hospital, Third Military Medical University, Chongqing 400042, China.; ^5^ College of Bioengineering, Chongqing University, Chongqing 400044, China.; ^6^ Department of Clinical Hematology, Faculty of Laboratory Medicine, Third Military Medical University, Chongqing 400038, China.

## Abstract

Osteoarthritic cartilage tissue displays a characteristic imbalance in trace element metabolism closely associated with the production of oxidative stress. This study revealed the pivotal role of iron-overload-triggered and selenoprotein-catalyzed lipid peroxidation in osteoarthritis pathogenesis. Based on this discovery, we innovatively pioneered the integration of Fe^2+^-capturing (attack) nanocatalysts and selenium-enriched (defense) polypeptides (selenomethionine [SeMet]) into a hydrogel platform. Polyvinylpyrrolidone-assembled magnesium hexacyanoferrate (MgHCF) nanoparticles enabled efficient Fe^2+^ chelation to counteract ferroptosis. SeMet utilization by the selenoprotein glutathione peroxidase 4 is required to prevent hydroperoxide-induced ferroptosis. Furthermore, a dynamically cross-linked network was constructed from oxidized hyaluronic acid (OHA) and hyaluronic acid–adipic acid dihydrazide (HA-ADH), enhanced by grafting SeMet onto the OHA chain and encapsulating MgHCF into the polymeric matrix. MgHCF@OHA/HA-ADH/SeMet hydrogels exhibited injectable, self-healing, and sustainable drug release capabilities. The platform promoted the anti-lipid peroxidation process and restored the mitochondrial homeostasis of chondrocytes under inflammatory stimulation via the phosphoinositide 3-kinase/Akt/forkhead box O1 pathway. In vivo investigations showed its anti-inflammatory activity, ferroptosis-inhibiting capacity, and ability to reverse cartilage degeneration. This work establishes a therapeutic paradigm based on trace element synergy, providing a translatable disease-modifying candidate.

## Introduction

Catalytic antioxidation is a promising frontier in biomedical research, targeting the reversal of complex pathological processes such as cancer, inflammation, and metabolic degeneration [1,2]. A major driver of these processes is oxidative stress, triggered by reactive oxygen species (ROS) and/or metal ion accumulation [3–5]. Such oxidative stress, together with associated transcriptional and proteomic alterations, disrupts cellular and tissue function, contributing to diverse diseases [2]. Increasing evidence shows that redox-imbalance-induced oxidative stress in chondrocytes critically impairs cartilage homeostasis in osteoarthritis (OA), a chronic degenerative disease affecting over 22% of individuals above 40 years and imposing a huge socioeconomic burden [6,7]. Thus, elucidating OA pathogenesis is essential for developing safe and effective therapies.

Trace elements are now understood to have a vital function in regulating redox processes within cells, serving as essential cofactors for normal cell growth and survival. However, recent pioneering investigations have illustrated that osteoarthritic cartilage tissue displays a characteristic imbalance in trace element metabolism, along with the production of oxidative stress [8–10]. Importantly, this finding highlights the chondrocytic pathological origin of trace element imbalance, along with secondary oxidative stress, as a substantial therapeutic target for restoring trace element homeostasis and potentially preventing OA. During long-term joint remodeling, the joint adapts to specific levels of trace elements, including iron, copper, zinc, and selenium. Any deviation from these optimal levels—whether excess or deficiency—can impair joint function and predispose the joint to OA. Furthermore, recent studies suggest that disruptions in the redox system within OA chondrocytes, coupled with iron deposition, contribute to the occurrence of ferroptosis [11,12]. In contrast, our previous research identified specific alterations in selenoproteins within OA chondrocytes. Interventions aimed at rebalancing selenium levels have shown promise in maintaining cartilage homeostasis by targeting selenoproteins to scavenge ROS [13]. Notably, the identification of ferroptosis, along with the roles of iron and selenium, underscored the critical importance of intracellular trace element homeostasis [14]. Iron homeostasis and selenium metabolism intersect in the study of ferroptosis, a nonapoptotic type of programmed cell death that is strictly related to lipid peroxidation in cartilage and accelerates the progression of OA [15,16]. Given these findings, there is clinical interest in developing biomaterials capable of simultaneously decreasing lipid peroxidation levels resulting from iron-mediated cell death and improving selenoprotein expression. Such strategies could emerge as vital therapeutic approaches for improving ferroptosis-triggered OA.

Ferric hexacyanoferrate (Fe_4_[Fe(CN)_6_]_3_), also known as Prussian blue (PB), received approval from the US Food and Drug Administration as a detoxification agent for radioactive cesium and thallium poisoning, owing to its proven in vivo biocompatibility and biosafety [17,18]. Beyond this traditional use, PB nanoparticles have been engineered into diverse structures as nanotherapeutics for drug delivery and antioxidation [19]. Moreover, PB analogs, synthesized by substituting non-iron species for ferrous and/or ferric ions, have emerged as multifunctional nanotherapeutics [20]. PB analog nanoparticles possess a strong iron-chelating capacity, attributed to the extremely low solubility product constant of iron-based PB (3.3 × 10^−41^), which is lower than that of non-iron analogs. This enables efficient ferrous ion capture through rapid re-formation of PB nanoparticles [21,22], highlighting their broad potential in biomedical applications.

All selenium within selenoproteins is located in active centers such as Sec, enabling them to perform physiological functions, such as antioxidant, anti-inflammatory, and osteogenic effects [23]. Among selenium-based compounds, selenomethionine (SeMet) can elevate selenium levels and be utilized in the synthesis of glutathione peroxidase (GPX) proteins. This process rapidly replenishes the selenium necessary for the catalytic functions of GPXs without the need for complex transformation pathways [24]. Previous studies have demonstrated that selenium utilization by glutathione peroxidase 4 (GPX4) is required to prevent hydroperoxide-induced ferroptosis [25,26]. However, small amino acid molecules such as SeMet face limitations, including low stability, poor bioavailability, and a short half-life, which can compromise their biological activity over time [27,28]. These challenges may restrict the broader application of SeMet in cartilage regeneration. Therefore, developing a delivery system that can sustain releasing highly active amino acid molecules remains a critical issue that needs to be solved.

Over recent decades, synthetic hydrogels—hydrophilic polymer networks resembling biological tissues—have advanced in drug delivery [28–30]. Their biocompatibility, tunable biodegradability, and controllable mechanical strength [31,32] have enabled widespread application in OA therapy. However, conventional hydrogels are limited by single functionality, low mechanical strength, elevated porosity (resulting in rapid erosion and drug diffusion), and uncontrolled drug release in pathological conditions, restricting further development. Hyaluronic acid (HA)-based hydrogels, in particular, provide abundant reactive sites and distinct biological functions [33,34]. Indeed, small amino acid molecules offer advantages due to their structural diversity, defined configurations, ease of modification, and low immunogenicity [35,36]. Accordingly, this study employed cross-linked HA hydrogels as a foundational template. By establishing covalent bonds between the hydrogel and amino acid molecules, such as SeMet, through the amino or carboxyl termini of peptides, a dynamically modified hydrogel system can be engineered. This approach ensured both high bioactivity and the sustained release of amino acid molecules.

**Fig. 1.**
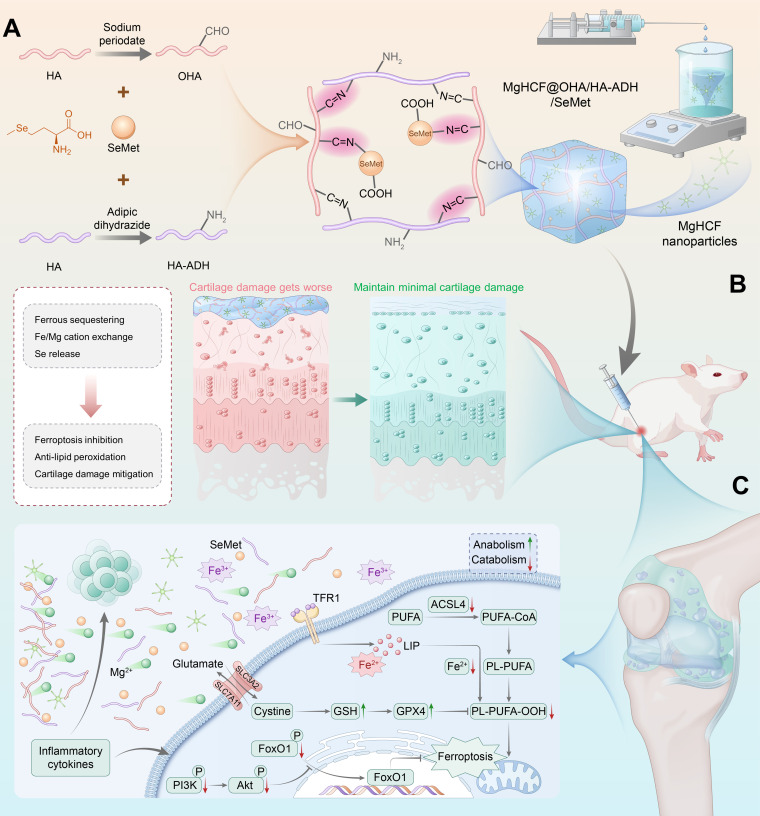
Schematic illustration of the preparation procedure of the MgHCF@OHA/HA-ADH/SeMet hydrogel and its antiferroptosis mechanism capable of “attack and defense” to combat osteoarthritis (OA). (A) The fabrication process of the MgHCF@OHA/HA-ADH/SeMet hydrogel for ferrous sequestering and catalytic anti-lipid peroxidation. (B) Injectable cross-linked MgHCF@OHA/HA-ADH/SeMet hydrogels developed as an intra-articular delivery platform for sustainably releasing MgHCF and SeMet in the inflamed joint. (C) Schematics of the mechanism of the MgHCF@OHA/HA-ADH/SeMet hydrogel for osteoarthritic cartilage damage mitigation. MgHCF, magnesium hexacyanoferrate; OHA, oxidized hyaluronic acid; HA-ADH, hyaluronic acid–adipic acid dihydrazide; SeMet, selenomethionine; TFR1, transferrin receptor protein 1; SLC3A2, solute carrier family 3 member 2; SLC7A11, solute carrier family 7 member 11; LIP, labile iron pool; PL, phospholipids; ACSL4, acyl-CoA synthetase long-chain family member 4; PUFA, polyunsaturated fatty acid; GSH, glutathione; GPX4, glutathione peroxidase 4; FoxO1, forkhead box O1; PI3K, phosphoinositide 3-kinase.

Here, microarray transcriptional profiling analysis highlights the dysregulation of iron metabolism and selenoprotein-catalyzed lipid peroxidation during cartilage degeneration. In light of the above considerations, we innovatively pioneered the integration of Fe^2+^-capturing magnesium hexacyanoferrate (MgHCF) nanoparticles, and selenium-enriched SeMet into an oxidized hyaluronic acid (OHA)/hyaluronic acid–adipic acid dihydrazide (HA-ADH) hydrogel platform. A schematic illustration of the fabrication of the injectable hydrogel system and the main research idea of this study is presented in Fig. 1A to C. Based on the results, we (a) show that MgHCF@OHA/HA-ADH/SeMet hydrogels exhibit injectable, self-healing, and sustainable drug release capabilities; (b) verify that MgHCF@OHA/HA-ADH/SeMet hydrogels work synergistically to mitigate ferroptosis-associated cartilage damage via the phosphoinositide 3-kinase (PI3K)/Akt/forkhead box O1 (FoxO1) signaling pathway; and (c) confirm that MgHCF@OHA/HA-ADH/SeMet hydrogels mitigate inflammatory responses, extracellular matrix remodeling, and ferroptosis when injected intra-articularly into OA rats.

## Results

### Ferroptosis-related molecular patterns linked to iron metabolism dysregulation and lipid peroxidation resistance in OA cartilage

**Fig. 2.**
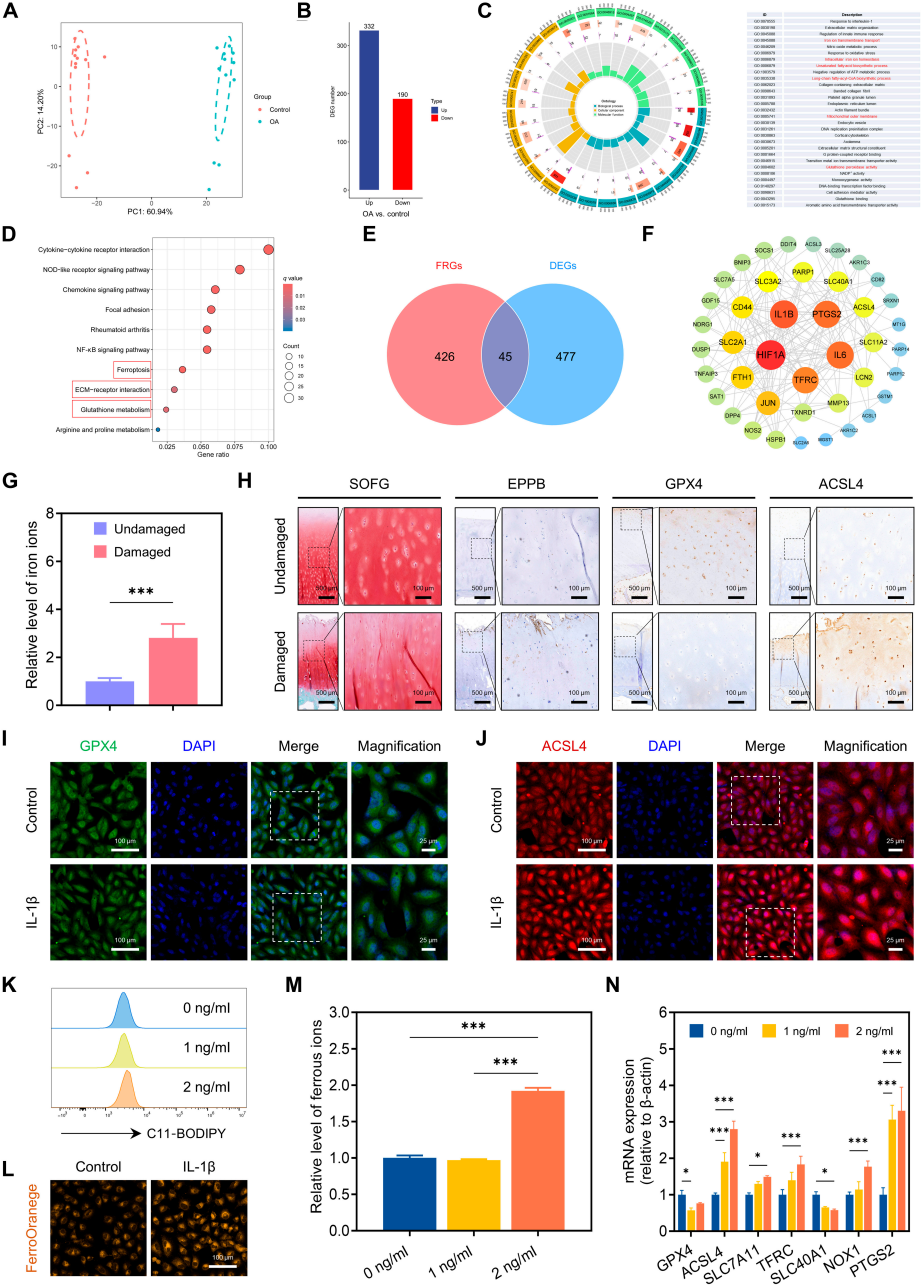
Ferroptosis-related molecular patterns linked to iron metabolism dysregulation and lipid peroxidation resistance in OA cartilage. (A) Principal component analysis (PCA) of differentially expressed genes (DEGs) in the 2 groups (*n* = 12). (B) Numbers of DEGs in the 2 groups. (C) The enriched Gene Ontology (GO) terms for DEGs between human OA samples and control samples based on 3 sub-ontologies: biological process, cellular component, and molecular function. (D) Kyoto Encyclopedia of Genes and Genomes (KEGG) analysis of the signaling pathway enriched by the DEGs between human OA samples and control samples. (E) Forty-five ferroptosis-related DEGs (FRDEGs) from the intersection of DEGs and ferroptosis-related genes (FRGs). (F) Protein–protein interaction (PPI) network of hub genes among human FRDEGs. (G) Relative level of iron ions in human OA cartilage and corresponding nonlesion cartilage (*n* = 4). (H) Cartilage sections from the undamaged or damaged region of human OA cartilage were stained with Safranin O–Fast Green (SOFG) and enhanced Perls’ Prussian blue and immunostained against GPX4 and ACSL4. Scale bar, 500 μm. Magnification: scale bar, 100 μm. Immunofluorescence staining of the (I) GPX4 and (J) ACSL4 levels of chondrocytes in the absence or presence of interleukin-1β (IL-1β). Scale bar, 100 μm. Magnification: scale bar, 25 μm. (K) Fluorescence-activated cell sorting analysis of C11-BODIPY fluorescence (*n* = 3). (L) The FerroOrange probe was used to quantify the level of Fe^2+^ expression in chondrocytes. Scale bar, 100 μm. (M) Relative level of Fe^2+^ content in chondrocytes treated with different concentrations of IL-1β (*n* = 3). (N) Polymerase chain reaction (PCR) with reverse transcription detection of *GPX4*, *ACSL4*, *SLC7A11*, *TFRC*, *SLC40A1*, *NOX1*, and *PTGS2* expression in chondrocytes (*n* = 3). Data are represented as mean ± SD. *P < 0.05. ATP, adenosine triphosphate; NF-κB, nuclear factor kappa B; ECM, extracellular matrix; DAPI, 4′,6-diamidino-2-phenylindole; mRNA, messenger RNA.

The human OA dataset GSE75181 was obtained from the Gene Expression Omnibus database to assess the aberrant metabolic signature related to degenerative cartilage. Principal component analysis revealed a clear distinction between the transcriptional profiles of the OA and control groups, highlighting variations in gene expression patterns (Fig. 2A). At a significance threshold of *P* < 0.05, 522 differentially expressed genes (DEGs) were determined, including 332 overexpressed genes and 190 suppressed genes, as illustrated in the volcano plot (Fig. 2B and Fig. S1A and B). Gene Ontology (GO) enrichment analysis illustrated that the majority of DEGs were strongly associated with biological processes, including iron ion transmembrane transport, intracellular iron ion homeostasis, unsaturated fatty acid biosynthesis, and long-chain fatty-acyl-CoA biosynthesis. Within the cellular component category of GO, the top-ranking terms included the collagen-containing extracellular matrix (ECM) and the mitochondrial outer membrane, suggesting their potential involvement in OA pathology. In terms of molecular function, the most enriched GO terms were GPX activity, NADP^+^ activity, and glutathione (GSH) binding (Fig. 2C). Moreover, Kyoto Encyclopedia of Genes and Genomes (KEGG) pathway analysis of the DEGs illustrated enrichment in pathways such as ferroptosis, ECM–receptor interaction, and GSH metabolism (Fig. 2D). These bioinformatics analyses collectively suggested that the OA onset and progression are closely linked to cellular iron metabolism, lipid homeostasis, and ferroptosis. This was further supported by gene set enrichment analysis, which established positive enrichment of genes related to “ferroptosis” and “arachidonic acid metabolism” in the transcriptome of OA chondrocytes (Fig. S2). Subsequently, 512 ferroptosis-related genes (FRGs) were determined from the FerrDb database. Among these, 45 ferroptosis-related DEGs (FRDEGs) were detected through the intersection of DEGs and FRGs (Fig. 2E). GO and KEGG enrichment analyses of these FRDEGs illustrated their involvement in biological processes (including responses to iron ion transport and fatty acid metabolism) as well as molecular functions (very long-chain fatty acid-CoA ligase activity and ferrous iron binding). KEGG pathway analysis further indicated that FRDEGs were related to ferroptosis, fatty acid, central carbon, and GSH metabolism (Fig. S3A and B). To examine the interactions among these genes, a protein–protein interaction network of the 45 FRDEGs was produced using the STRING database and visualized in Cytoscape. The cytoHubba plug-in was employed to identify hub genes using the degree algorithm. Based on correlation coefficients, key FRGs, such as *TFRC*, *IL1B*, *IL6*, *PTGS2*, *ACSL4*, and *SLC40A1*, were identified as having high degrees of connectivity within the network (Fig. 2F). In summary, these outcomes illustrate that both iron metabolism and lipid peroxidation play roles in OA pathogenesis.

Building on the bioinformatics analysis presented earlier, we measured the levels of iron ions in both undamaged and damaged cartilage tissues from OA patients. The results demonstrated that the total iron ion levels were higher in damaged cartilage than in undamaged cartilage (Fig. 2G). Furthermore, iron deposition, as visualized by enhanced Perls’ Prussian blue staining, was detectable in damaged cartilage (Fig. 2H), indicating iron accumulation in cartilage during OA progression. To further explore the molecular mechanisms underlying ferroptosis in OA, we investigated ferroptosis-related molecular patterns in both undamaged and damaged cartilage tissues. As a selenoprotein, GPX4 has a critical function in eliminating lipid peroxides and maintaining the antioxidant defense system and is known to serve as a central regulator of antiferroptosis [37]. In our study, GPX4 was strongly expressed in intact regions of arthritic cartilage but was nearly absent in OA-affected areas. In contrast, acyl-CoA synthetase long-chain family member 4 (ACSL4)—which catalyzes polyunsaturated fatty acid (PUFA)-containing lipid synthesis and drives lipid peroxidation accumulation [38]—was markedly elevated in OA-damaged cartilage (Fig. 2H). Consistent with these findings, GPX4 and ACSL4 protein expression in human cartilage samples was down-regulated and upregulated, respectively, at both the tissue and cellular levels (Fig. 2I and J). To assess lipid peroxidation, we employed C11-BODIPY, a fluorescent probe widely used in ferroptosis studies, and quantified intracellular lipid ROS levels using flow cytometry. Figure 2K illustrates that the relative levels of lipid ROS increased progressively with higher concentrations of interleukin-1β (IL-1β). Given that ferroptotic cell death is associated with the accumulation of cellular Fe^2+^ [39], we also analyzed Fe^2+^ levels and observed an elevation in the abnormal buildup of Fe^2+^ after IL-1β treatment compared to the that in the control group (Fig. 2L). Moreover, we measured the ferrous ion content in C28/I2 chondrocytes after incubation with several IL-1β levels and found that it increased approximately 2-fold under 2 ng/ml IL-1β stimulation compared to that in the 0 ng/ml group (Fig. 2M). Depending on KEGG pathway and GO enrichment analyses (Fig. S4), we identified altered expression of genes related to iron metabolism and lipid peroxidation in OA chondrocytes, including *SLC40A1*, *TFRC*, *ACSL4*, and *PTGS2*. Quantitative polymerase chain reaction analysis further demonstrated that the levels of *ACSL4*, *SLC7A11*, *TFRC*, *NOX1*, and *PTGS2* were markedly increased under IL-1β stimulation and exhibited dose-dependent elevation. In contrast, treatment with IL-1β decreased the levels of *GPX4* and *SLC40A1* (Fig. 2N), rendering the cells more sensitive to ferroptosis. In summary, these findings provide compelling evidence that dysregulation of iron metabolism and impaired resistance to lipid peroxidation are key drivers of OA progression. In light of the above observations, biomaterials capable of cooperatively decreasing lipid peroxidation levels and mitigating iron-induced cell death could emerge as vital therapeutic approaches for alleviating ferroptosis-triggered OA.

### Fabrication and characterization of MgHCF nanoparticles and MgHCF@OHA/HA-ADH/SeMet hydrogels

**Fig. 3.**
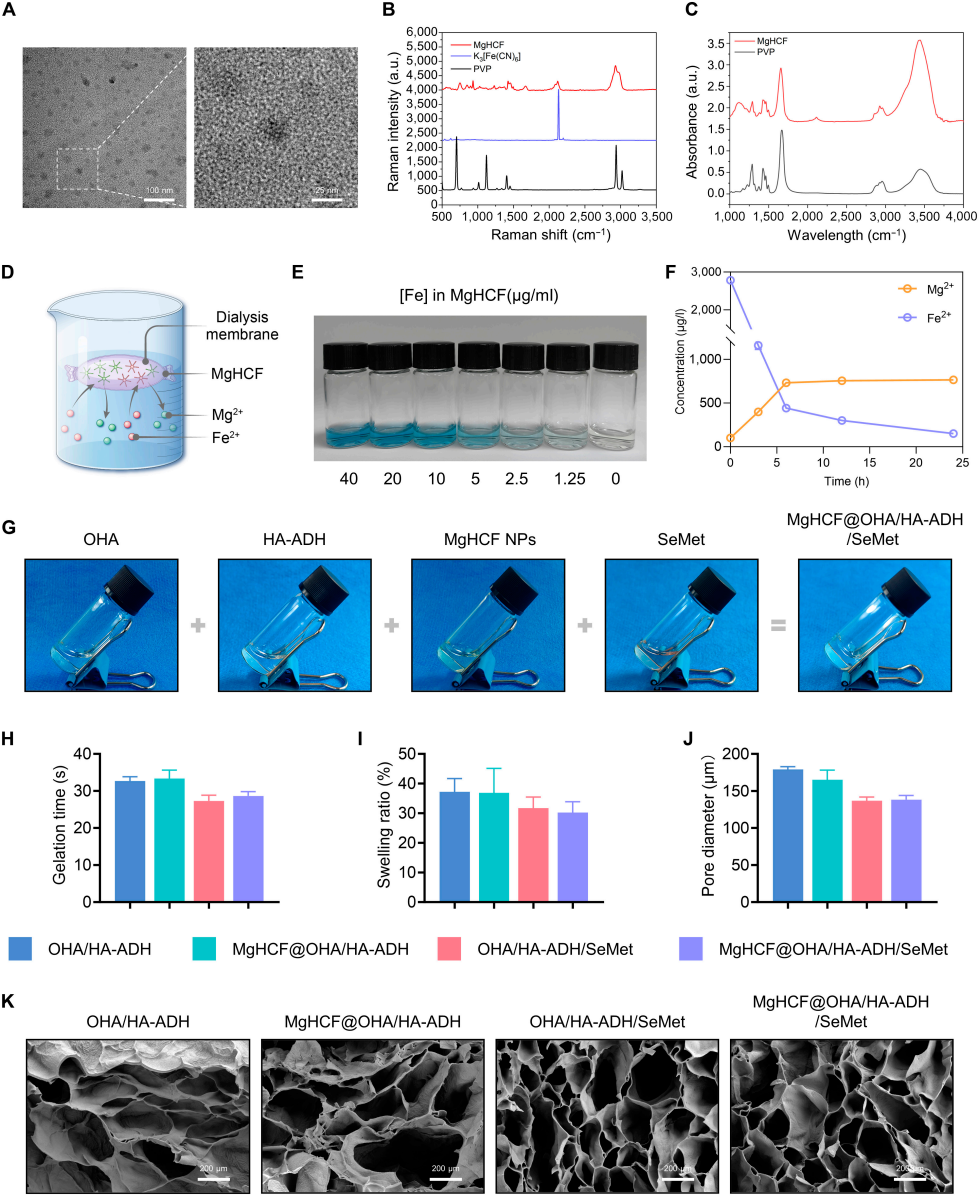
Synthesis of MgHCF@OHA/HA-ADH/SeMet hydrogels. (A) Transmission electron microscopy (TEM) image of MgHCF nanoparticles. Scale bar, 100 nm. The boxed area is enlarged on the right; the scale bar is 25 nm. (B) Raman spectra and (C) Fourier transform infrared spectroscopy (FTIR) spectra of MgHCF nanoparticles, polyvinylpyrrolidone (PVP), and K_3_[Fe(CN)_6_]. (D) Schematic illustration of the dialysis-membrane separation-regulated cation exchange for MgHCF nanoparticles. (E) Digital photograph of the aqueous solutions containing MgHCF nanoparticles with the additions of ferrous ions at varying concentrations. (F) Time-dependent elemental concentration profiles of Mg^2+^ and Fe^2+^ during the dialysis process. (G) The gelation process images of MgHCF@OHA/HA-ADH/SeMet hydrogels. (H) Gelation time, (I) swelling ratio, and (J) pore diameter of the hydrogel samples. (K) Scanning electron microscopy (SEM) analysis of the lyophilized hydrogel structure. Scale bar, 200 μm. NPs, nanoparticles.

Recent pioneering investigations have illustrated that a characteristic imbalance in trace element (iron and selenium) metabolism is involved in lipid peroxidation [40–42]. We attempted to construct a trace element modulated biomaterial to attenuate lipid peroxidation and reverse the degenerative state of articular cartilage. The preparation of MgHCF nanoparticles was conducted following previously reported literature with slight modifications [43]. The morphologies of MgHCF nanoparticles were distinguished via transmission electron microscopy, which illustrated that the nanoparticles exhibited excellent dispersion and maintained uniform spherical shapes (Fig. 3A and Fig. S5A). Energy-dispersive spectrometry mapping illustrated the uniform distribution of Mg and Fe in MgHCF nanoparticles (Fig. S5B). Dynamic light scattering analysis indicated an average nanoparticle size of approximately 40 nm, indicating that the synthesized MgHCF nanoparticles were of high purity and relatively homogeneous in both structure and size (Fig. S5C). X-ray diffraction analysis illustrated the characteristic peaks of the polyvinylpyrrolidone (PVP) polymer in MgHCF powder (Fig. S5D). Raman spectroscopy of MgHCF nanoparticles showed PVP-specific Raman shifts between 500 and 1,800 cm^−1^ and a peak at 2,132 cm^−1^ corresponding to K_3_[Fe(CN)_6_] (Fig. 3B) [44]. Fourier transform infrared spectroscopy further confirmed C≡N stretching vibrations in MgHCF (Fig. 3C). Together, these results verified the successful synthesis of MgHCF nanoparticles. The nanoparticles could exhibit strong ferrous ion affinity, promoting the re-formation of the original PB nanostructure while releasing Mg^2+^ (Fig. 3D). To assess cation exchange with ferrous ions, different concentrations of ferrous sulfate (0 to 40 μg/ml) were added to MgHCF suspensions (40 μg/ml), producing immediate optical absorption changes and a color shift from green to blue. The observation of a blue color and the absence of green indicated that Fe^2+^ exchanged with the Mg^2+^ present (Fig. 3E). Ionic dynamics were further examined by immersing MgHCF nanoparticles (1 mg/ml, 10 ml) in a dialysis membrane within ferrous sulfate solution (20 μg/ml). At predetermined time points, the concentration of iron decreased, while the concentration of magnesium increased, consistent with a displacement reaction (Fig. 3F).

The successful preparation of HA-ADH, OHA, and OHA/SeMet was confirmed by Fourier transform infrared spectroscopy and ^1^H nuclear magnetic resonance spectral analysis (Fig. S6A and B). Cell Counting Kit-8 (CCK-8) assay results demonstrated that groups treated with MgHCF or SeMet at a concentration of ≤80 μg/ml or ≤30 μM, respectively, exhibited high cell viability, approaching 100% of that of the 0 μM group (Fig. S7A and B). Composite hydrogels, namely, MgHCF@OHA/HA-ADH, OHA/HA-ADH/SeMet, and MgHCF@OHA/HA-ADH/SeMet, were obtained by incorporating SeMet and MgHCF during the dynamic hydrogel formation process (Fig. 3G). Comparative analysis illustrated that adding SeMet and MgHCF had no effect on the gelation time (Fig. 3H). Figure 3I illustrates that all hydrogels displayed slightly lower swelling ratios compared to pure HA hydrogels, with MgHCF@OHA/HA-ADH/SeMet showing the lowest swelling ratio at 30.22% ± 3.65%. Lyophilized hydrogel samples, including OHA/HA-ADH, MgHCF@OHAHA-ADH, OHA/HA-ADH/SeMet, and MgHCF@OHA/HA-ADH/SeMet, displayed microporous structures with mean pore sizes of 37.19 ± 4.51, 36.84 ± 8.27, 31.70 ± 3.76, and 30.22 ± 3.65 μm, respectively (Fig. 3J and K). Taken together, these outcomes illustrate that the incorporation of SeMet could result in a denser hydrogel network, likely due to the formation of new dynamic bonds.

**Fig. 4.**
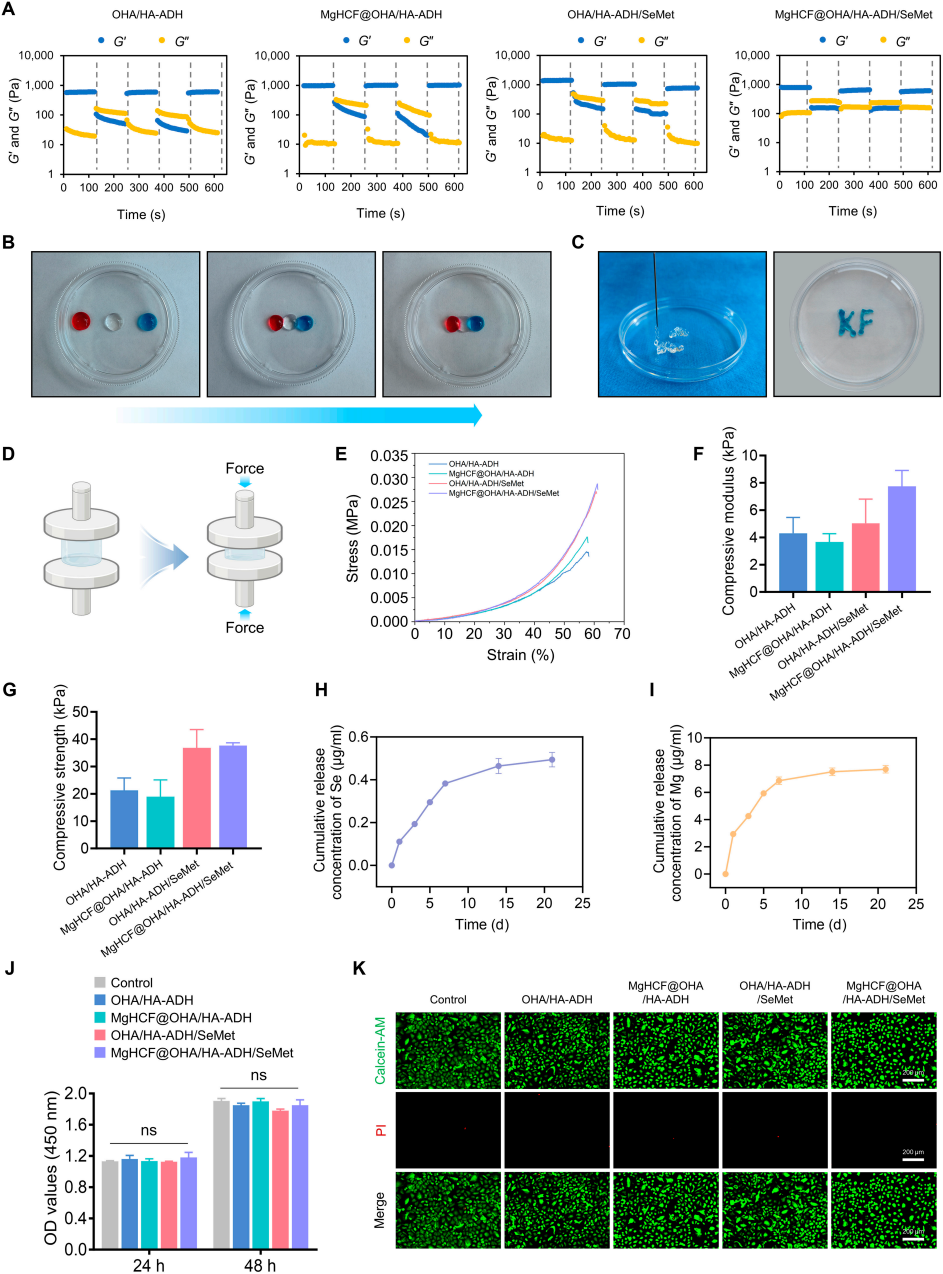
Characterization of MgHCF@OHA/HA-ADH/SeMet hydrogels. (A) Rheological behaviors of the hydrogel with alternate strains switched from 1% to 100% for 3 cycles. (B) Photographs of the macroscopic healing capacities of the hydrogel. (C) Images of MgHCF@OHA/HA-ADH/SeMet hydrogels injected from a 26-gauge needle without clogging. (D) Illustration of the compression test. Created with BioRender.com. (E) Compression curves of the hydrogels. (F) The compression modulus and (G) compression strength of the hydrogels. The release performance of (H) Se and (I) Mg from the MgHCF@OHA/HA-ADH/SeMet hydrogel. (J) Cell Counting Kit-8 (CCK-8) test and (K) Live/Dead staining of C28/I2 cells treated by the hydrogels (*n* = 3). Data are represented as mean ± SD. ns means no significance. OD, optical density; PI, propidium iodide.

The storage modulus (*G*′) increased upon the addition of SeMet into the hydrogel, which could be attributed to the rise in cross-linking density within the hydrogel network. The strain sweep results revealed that the critical point, illustrating the collapse of the hydrogel network, occurred when *G*′ and *G*″ intersected at approximately 100% strain (Fig. S8). Based on this finding, a continuous alternating strain sweep was conducted. The oscillatory strain amplitudes were alternated between 1% and 100%, with each interval lasting 120 s. Under increased dynamic strain, *G*′ reduced and fell below *G*″. However, when the strain was reduced to a lower value, both *G*′ and *G*″ promptly returned to their original values in all cycles (Fig. 4A). Furthermore, 3 separate hydrogels dyed pink, transparent, and blue were combined into a single, cohesive hydrogel, demonstrating excellent self-healing properties (Fig. 4B). The hydrogel could be continuously injected without clogging through a 26-gauge needle (Fig. 4C). After injection into a joint cavity, the dynamic hydrogel is subjected to various mechanical stresses, necessitating adequate resistance to compression. Therefore, the compressive properties of the dynamic hydrogels were assessed (Fig. 4D). The compressive results were consistent with the rheological results, and the stress–strain curve showed the nonlinear mechanical behavior of the hydrogels under compression (Fig. 4E). Compared with that of OHA/HA-ADH, the compressive modulus of MgHCF@OHA/HA-ADH did not exhibit a change, while the introduction of SeMet caused an increase, with values reaching 5.04 ± 1.77 kPa for OHA/HA-ADH/SeMet and 7.75 ± 1.16 kPa for MgHCF@OHA/HA-ADH/SeMet (Fig. 4F). The compressive strength followed a similar trend to the compressive modulus (Fig. 4G).

Next, the hydrogel degradation rate was evaluated in phosphate-buffered saline (PBS). Among the tested hydrogels, the OHA/HA-ADH hydrogel exhibited the fastest degradation rate, with only 1.39% of its mass remaining after a 7-d incubation. In contrast, the residual weights of the MgHCF@OHA/HA-ADH, OHA/HA-ADH/SeMet, and MgHCF@OHA/HA-ADH/SeMet hydrogels were 4.63%, 15.22%, and 18.23%, respectively (Fig. S9). Moreover, as shown in Fig. 4H and I, selenium and magnesium were released in a sustained manner over 21 d as the MgHCF@OHA/HA-ADH/SeMet hydrogel degraded. These results suggested that the hydrogel remained relatively stable under physiological conditions. However, the pH-responsive Schiff base linkers could be cleaved in an OA microenvironment, enabling the on-demand release of SeMet and MgHCF nanoparticles. Furthermore, MgHCF@OHA/HA-ADH/SeMet hydrogel biocompatibility was assessed via the Live/Dead staining and CCK-8 assay. The CCK-8 outcomes illustrated that the cell count in each group elevated over time, with no variations observed between the groups (Fig. 4J). Likewise, the Live/Dead assessment outcomes illustrated that nearly all cells remained viable after 2 d of culture (Fig. 4K). The above outcomes illustrate the excellent biocompatibility of MgHCF@OHA/HA-ADH/SeMet hydrogels.

### In vitro therapeutic effects of MgHCF@OHA/HA-ADH/SeMet hydrogels

**Fig. 5.**
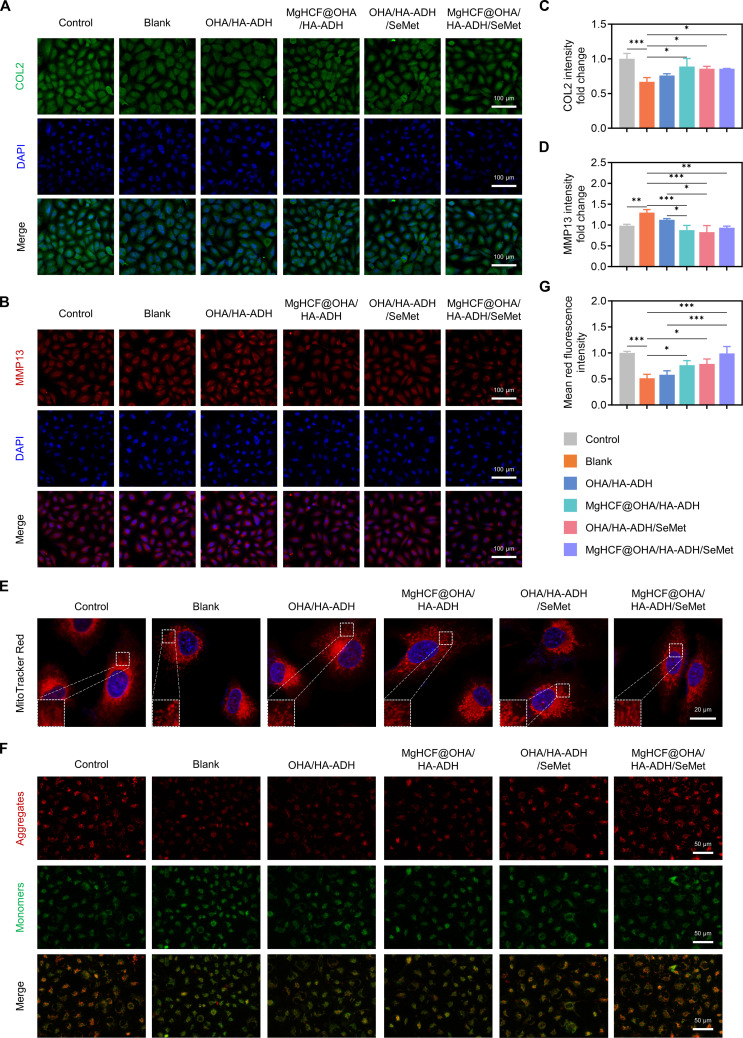
Assessment of MgHCF@OHA/HA-ADH/SeMet hydrogels on matrix restoration and mitochondrial homeostasis. (A to D) Immunofluorescence images of collagen type II (COL2; green) and matrix metallopeptidase 13 (MMP13; red) after incubation with each group and corresponding relative fluorescence intensity quantification (*n* = 3). Scale bar, 100 μm. (E) Representative images of chondrocytes 48 h post indicated treatments visualized with MitoTracker Red staining. Dotted areas are magnified in the bottom left. Scale bar, 20 μm. (F) JC-1 staining and (G) quantification of chondrocytes following co-incubation with each group (*n* = 3). Scale bar, 50 μm. Data are represented as mean ± SD. *P < 0.05; **P < 0.01; ***P < 0.001.

As reported in previous studies, oxidative stress can trigger chondrocyte damage, leading to the biodegradation of the ECM. Moreover, the levels of matrix-degrading enzymes play a role in matrix degradation in OA cartilage [45]. Therefore, we investigated the expression of ECM-related markers in an IL-1β-stimulated human chondrocyte cell line (C28/I2) co-cultured with the leaching solution of different hydrogels. As shown in Fig. 5A to D, immunofluorescence analysis revealed that the MgHCF@OHA/HA-ADH/SeMet hydrogel could overexpress collagen type II (COL2) and suppress matrix metallopeptidase 13 (MMP13) in IL-1β-treated C28/I2 cells, suggesting that it effectively reversed ECM metabolic imbalance in degenerated chondrocytes. We subsequently investigated the effects of the MgHCF@OHA/HA-ADH/SeMet hydrogel on the dysregulation of IL-1β-triggered mitochondrial activity. Using MitoTracker Red staining, we observed morphological changes in C28/I2 chondrocytes following IL-1β treatment, characterized by fragmented and shrunken mitochondria. Importantly, treatment with the MgHCF@OHA/HA-ADH/SeMet hydrogel effectively mitigated the IL-1β-induced mitochondrial damage in C28/I2 chondrocytes (Fig. 5E). Mitochondrial membrane potential, a key indicator of cellular oxidative metabolic state [46], was also assessed. Posttreatment with the MgHCF@OHA/HA-ADH/SeMet hydrogel in the presence of IL-1β, the red fluorescence observed by the JC-1 probe was elevated compared to that of treatment with either the vehicle or OHA/HA-ADH alone (Fig. 5F and G). This increase in red fluorescence reflected an improved cellular energy state, suggesting a reduction in oxidative stress levels within the cells. In summary, these findings demonstrate that MgHCF@OHA/HA-ADH/SeMet hydrogels are capable of promoting ECM restoration and maintaining mitochondrial homeostasis through the synergistic release of SeMet and nanoparticles.

### Attack and defense therapy and in vitro antiferroptosis effect of MgHCF@OHA/HA-ADH/SeMet hydrogels

**Fig. 6.**
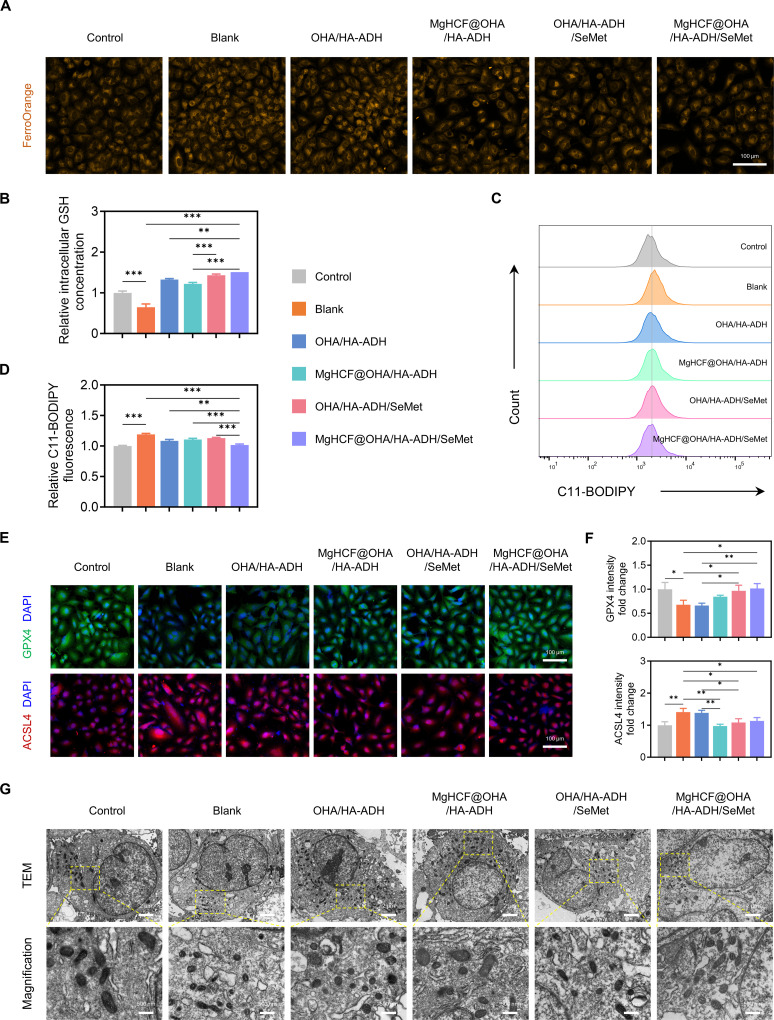
Efficacy of MgHCF@OHA/HA-ADH/SeMet hydrogels in ameliorating ferroptosis capable of attack and defense. (A) The FerroOrange probe was used to quantify the level of Fe^2+^ expression in chondrocytes. Scale bar, 100 μm. (B) Quantification of the relative concentration of GSH in chondrocytes (*n* = 3). (C) The cell lipid peroxidation signal was assayed via C11-BODIPY fluorescence by flow cytometry and (D) quantification (*n* = 3). (E) Immunofluorescence staining and (F) quantification of the GPX4 and ACSL4 levels in chondrocytes (*n* = 3). Scale bar, 100 μm. (G) TEM images of the indicated groups. Scale bars, 2 μm (low field), 500 nm (high field). Data are represented as mean ± SD. *P < 0.05; **P < 0.01; ***P < 0.001.

Lipid peroxidation and iron-dependent buildup characterize ferroptosis, a separate type of cell death. Many biological activities rely on it, including the metabolism of iron and PUFAs as well as the production of antioxidants like GSH [47]. Herein, we examined the antiferroptosis effects of the MgHCF@OHA/HA-ADH/SeMet hydrogel on C28/I2 chondrocytes following IL-1β treatment. To assess the accumulation of Fe^2+^ in chondrocytes, we utilized the FerroOrange probe. As illustrated in Fig. 6A, an increase in abnormal Fe^2+^ accumulation was observed in the IL-1β-treated group compared to that in the control group. However, Fe^2+^ accumulation was reduced to varying degrees following intervention with MgHCF@OHA/HA-ADH, OHA/HA-ADH/SeMet, and MgHCF@OHA/HA-ADH/SeMet. Notably, MgHCF@OHA/HA-ADH/SeMet demonstrated superior efficacy compared to the other 2 formulations. Given the disruption of cellular redox homeostasis during ferroptosis, these findings were further corroborated by the measurement of GSH concentrations across the different groups. Specifically, the relative GSH concentration in the MgHCF@OHA/HA-ADH/SeMet group increased compared to that in the blank group, suggesting that the hydrogel could effectively restore GSH production despite IL-1β-induced ferroptosis (Fig. 6B). In addition, we measured intracellular lipid ROS levels in C28/I2 cells treated with PBS, MgHCF@OHA/HA-ADH, OHA/HA-ADH/SeMet, or MgHCF@OHA/HA-ADH/SeMet hydrogels. C11-BODIPY staining illustrated that lipid peroxidation was elevated in response to IL-1β stimulation but was mitigated to varying degrees by the aforementioned interventions (Fig. 6C and D). Collectively, these findings indicate that MgHCF@OHA/HA-ADH/SeMet hydrogels ameliorate abnormal intracellular iron metabolism through their iron-chelating capacity and alleviate ferroptosis-related lipid peroxidation in chondrocytes under inflammatory conditions. The hydrogel’s antiferroptosis properties are likely attributable to the GPX4 activator SeMet and ferrous ion-capturing nanoparticles.

Subsequently, the role of MgHCF@OHA/HA-ADH/SeMet hydrogels in mitigating IL-1β-triggered ferroptosis in chondrocytes was assessed by enumerating key markers, GPX4 and ACSL4. As shown in Fig. 6E and F, immunofluorescence revealed that MgHCF@OHA/HA-ADH/SeMet exerted the strongest regulatory effects, upregulating GPX4 and down-regulating ACSL4 compared with those in the blank group. These results indicate that the hydrogels confer resistance to ferroptosis by enhancing ferroptosis-protective factors and suppressing pathogenic ones. Transmission electron microscopy analysis further demonstrated that IL-1β exposure induced mitochondrial atrophy and loss of cristae in C28/I2 cells, while these changes were alleviated by MgHCF@OHA/HA-ADH/SeMet treatment (Fig. 6G). Collectively, these findings suggest that MgHCF@OHA/HA-ADH/SeMet hydrogels attenuate inflammation-induced ferroptosis in chondrocytes through dual pharmacological actions.

### Transcriptomic and metabolomic profiling of MgHCF@OHA/HA-ADH/SeMet hydrogel-mediated cartilage protection

**Fig. 7.**
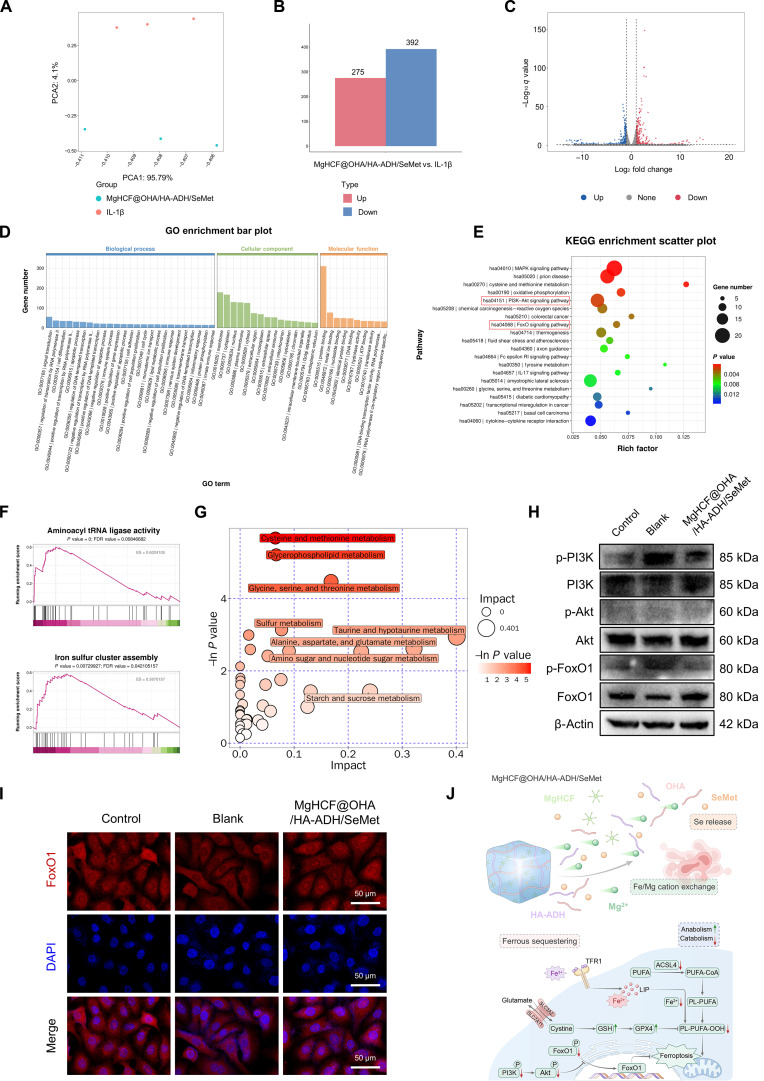
Transcriptomic and metabolomic shifts following MgHCF@OHA/HA-ADH/SeMet intervention in IL-1β-treated chondrocytes and therapeutic mechanism exploration of cartilage protection. (A) PCA of genes in chondrocytes after treatment with IL-1β or with IL-1β plus MgHCF@OHA/HA-ADH/SeMet. Three replicates are shown. (B) Numbers of DEGs in the 2 groups. (C) Volcano plots were generated between the IL-1β plus MgHCF@OHA/HA-ADH/SeMet group and the IL-1β group. (D) GO enrichment analysis of DEGs between the 2 groups. (E) KEGG enrichment analysis of the pathways involved in the biological effect induced by IL-1β or IL-1β plus MgHCF@OHA/HA-ADH/SeMet treatment. (F) Gene set enrichment analysis (GSEA) was applied to compare the gene sets involved in aminoacyl transfer RNA (tRNA) ligase activity and iron sulfur cluster assembly between the 2 groups. (G) Metabolic pathway enrichment results showing affected pathways, comparing the groups IL-1β plus MgHCF@OHA/HA-ADH/SeMet versus IL-1β. (H) Western blot analysis of the expression of proteins related to the PI3K/Akt/FoxO1 signaling pathway. (I) Immunofluorescence assay depicting the location of FoxO1 in C28/I2 cells in each group. Scale bar, 50 μm. (J) The potential mechanisms of the trace element modulated nanocomposite hydrogel for cartilage matrix reconstruction. MAPK, mitogen-activated protein kinase; ES, enrichment score; FDR, false discovery rate.

An illuminating exploration of the mechanisms that steer C28/I2 cells’ fate was undertaken through RNA sequencing analysis of the cell–MgHCF@OHA/HA-ADH/SeMet hydrogel interaction. Principal component analysis showed a clear demarcation between all genes within the MgHCF@OHA/HA-ADH/SeMet group and those within the IL-1β group, underlining their distinct transcriptional profiles (Fig. 7A). A total of 667 DEGs were determined, comprising 392 down-regulated and 275 upregulated genes (Fig. 7B and C and Fig. S10). Functional enrichment analysis revealed involvement in lipid metabolism, inflammatory response, and cellular components such as mitochondria and extracellular space (Fig. 7D). KEGG pathway analysis (bubble chart, Fig. 7E) highlighted enrichment in PI3K–Akt and FoxO signaling, cysteine and methionine metabolism, and oxidative phosphorylation. Heatmap analysis of PI3K–Akt-related genes showed that MgHCF@OHA/HA-ADH/SeMet upregulated PCK2, LAMC2, and CREB5 while down-regulating BCL2L11, PIK3R3, and NR4A1 (Fig. S11A). Clustering analysis of crucial genes related to the FoxO signaling pathway revealed that MgHCF@OHA/HA-ADH/SeMet down-regulated the expression of the genes, including TNFSF10, SKP2, BCL2L11, and PIK3R3 (Fig. S11B). Gene set enrichment analysis was employed to observe the influence of MgHCF@OHA/HA-ADH/SeMet treatment on various cellular processes. Consistently, the IL-1β plus MgHCF@OHA/HA-ADH/SeMet group displayed overexpression of genes participating in aminoacyl transfer RNA ligase activity and iron sulfur cluster assembly (Fig. 7F). The Cytoscape software was utilized to visualize the genes in the above pathways and generate a “pathwaytarget” diagram (Fig. S12). To further investigate intracellular metabolic changes induced by MgHCF@OHA/HA-ADH/SeMet and elucidate downstream pathways, untargeted metabolomics was performed on C28/I2 cells with or without MgHCF@OHA/HA-ADH/SeMet treatment. Pathway enrichment confirmed that cysteine and methionine metabolism, glycerophospholipid metabolism, and glycine, serine, and threonine metabolism were enriched in IL-1β plus MgHCF@OHA/HA-ADH/SeMet-treated cells compared with those in IL-1β-stimulated cells (Fig. 7G). Differentially abundant metabolites were further enriched in amino acid biosynthesis, sphingolipid metabolism, and carbon metabolism (Fig. S13). These outcomes illustrate that regulating the cartilage protection by MgHCF@OHA/HA-ADH/SeMet is potentially mediated by the PI3K/Akt/FoxO1 pathway, reprogramming ferroptosis-related patterns and thus reducing matrix degeneration.

To confirm the molecular mechanism of MgHCF@OHA/HA-ADH/SeMet hydrogel-mediated cartilage protection, we induced C28/I2 cells with IL-1β and MgHCF@OHA/HA-ADH/SeMet for 48 h. Western blot was used to assess PI3K, p-PI3K, Akt, p-Akt, FoxO1, and p-FoxO1 protein expression. Western blot results confirmed that adding MgHCF@OHA/HA-ADH/SeMet to C28/I2 cells resulted in decreased protein levels of p-PI3K, p-Akt, and p-FoxO1, as well as increased protein level of FoxO1, validating the successful inactivation of the PI3K/Akt pathway and suppression of FoxO1 phosphorylation (Fig. 7H). We next explored whether the MgHCF@OHA/HA-ADH/SeMet hydrogel is essential for FoxO1 nuclear translocation. Immunofluorescence staining of FoxO1 revealed that upon IL-1β stimulation, FoxO1 transferred from the nucleus to the cytoplasm, while the MgHCF@OHA/HA-ADH/SeMet hydrogel promoted the translocation of FoxO1 protein from the cytoplasm to the nucleus (Fig. 7I). Previous studies have demonstrated that notopterol mitigate OA by inhibiting PI3K/Akt phosphorylation and enhancing antioxidant defenses through GSH and GPX4 upregulation, thereby preventing lipid peroxidation [48]. FoxO1 nuclear activity regulates the transcription of lipid catabolism and antioxidant response genes [49], while reduced NAD^+^ levels promote FoxO1 phosphorylation, leading to GPX4 inhibition and ferroptosis induction [50]. GSH, the key cellular antioxidant capable of inhibiting lipid peroxidation via the activity of the enzyme GPX4, is generated directly from the sulfur-containing amino acid cysteine and indirectly from methionine via the transsulfuration pathway [51]. Therefore, MgHCF@OHA/HA-ADH/SeMet hydrogels could promote the antioxidant process in the ferroptosis of chondrocytes under inflammatory stimulation, possibly by impairing PI3K/Akt signaling and suppressing FoxO1 phosphorylation, thereby maintaining cartilage metabolic homeostasis (Fig. 7J).

### In vivo radiological evaluation of intra-articular delivery using MgHCF@OHA/HA-ADH/SeMet hydrogels

**Fig. 8.**
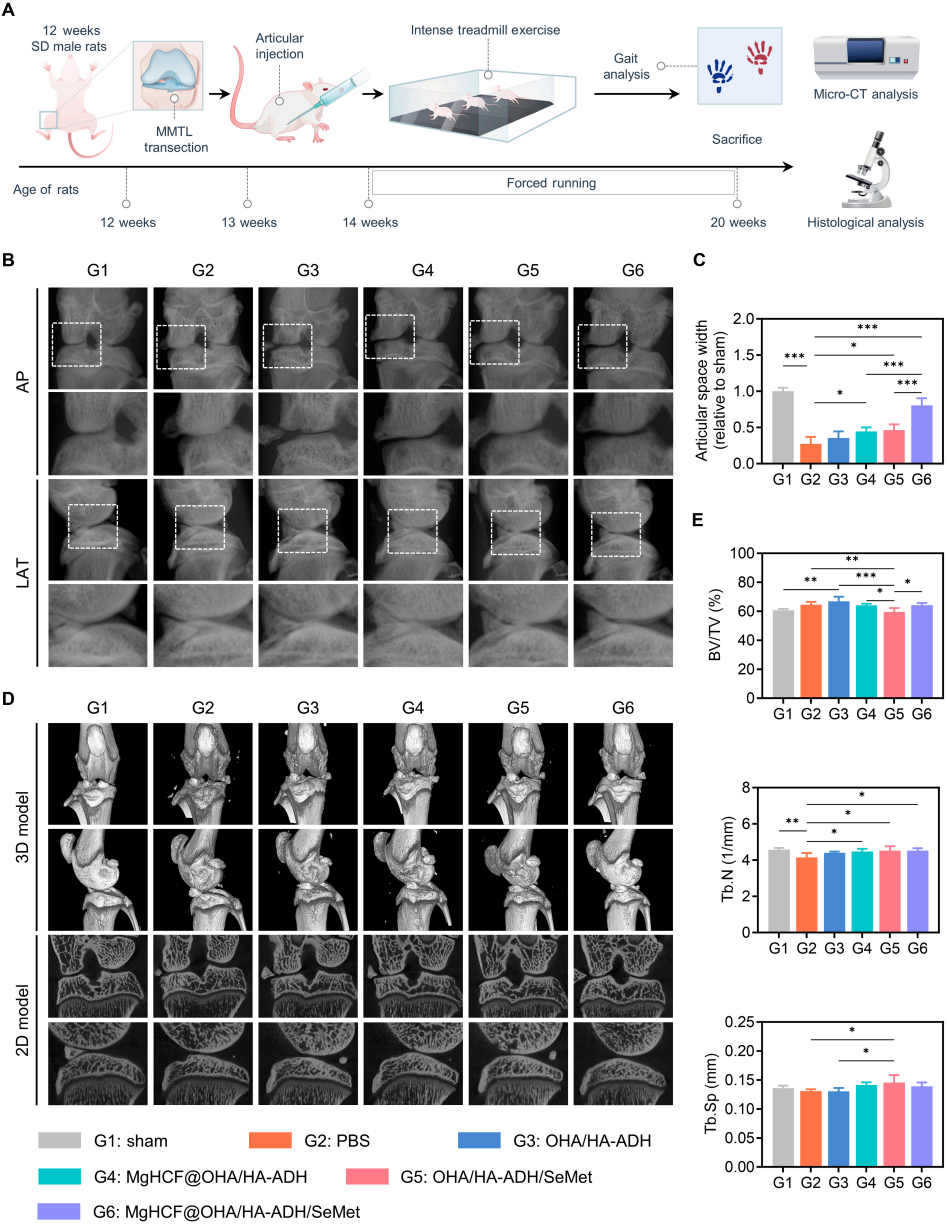
Radiological evaluation of osteoarthritic knee joints. (A) Schematic illustration of OA rat model construction induced by the combination of destabilization of the medial meniscus (DMM) with strenuous treadmill exercise, MgHCF@OHA/HA-ADH/SeMet hydrogel treatment, and therapeutic efficacy evaluation. SD, Sprague Dawley. (B) Radiographs of the knee joint in anteroposterior (AP) and lateral (LAT) views. (C) Eight weeks after surgery, the relative knee joint space width of the rat was quantified. (D) Representative 2-dimensional (2D) and 3-dimensional (3D) microcomputed tomography (micro-CT) images of the knee joints of rats in different groups. (E) Eight weeks after the operation, quantitative analysis of medial subchondral bone in rats. Data are computed as average ± SD (*n* = 5; “*”, “**”, and “***” represent statistical significance when setting the alpha level to 0.05, 0.01, and 0.001, respectively). MMTL, medial meniscotibial ligament; PBS, phosphate-buffered saline; BV/TV, bone volume/total volume; Tb.N, trabecular number; Tb.Sp, trabecular separation.

Based on the promising restorative effects observed on matrix degradation in vitro, we further evaluated the in vivo therapeutic efficacy of MgHCF@OHA/HA-ADH/SeMet hydrogels using a rat model of OA induced by surgical destabilization of the medial meniscus (DMM) combined with exercise intervention (Fig. 8A). At 8 weeks postsurgery, OA samples were collected from the knee joints and analyzed using x-ray imaging and microcomputed tomography (micro-CT). X-ray imaging revealed that the joint space width in the PBS-treated group was only 27.37% of that in the sham group, indicating joint space narrowing and structural destabilization following OA induction. In contrast, the hydrogel-treated groups showed varying degrees of joint space width recovery. Notably, the MgHCF@OHA/HA-ADH/SeMet group exhibited the most pronounced recovery, with the joint space width reaching up to 80.32% of that in the sham group, suggesting that the MgHCF@OHA/HA-ADH/SeMet hydrogel could effectively reverse joint space narrowing in the OA rat model. Furthermore, the joint space width in the MgHCF@OHA/HA-ADH and OHA/HA-ADH/SeMet groups was 1.26 times and 1.30 times greater, respectively, than that in the OHA/HA-ADH group (Fig. 8B and C). These results highlight the limited capacity of pure OHA/HA-ADH hydrogels to reverse joint space narrowing and underscore the synergistic roles of SeMet and MgHCF in enhancing cartilage repair and OA treatment efficacy. Furthermore, osteophyte formation in the hydrogel-treated groups followed a trend consistent with the recovery of joint space width, further demonstrating the ability of these composite hydrogels to delay OA progression (Fig. 8D). Micro-CT analysis revealed pathological changes in the subchondral bone, such as trabecular bone sclerosis and thickening, in the PBS and OHA/HA-ADH groups. These changes were alleviated to some extent in the other hydrogel-treated groups. Importantly, the MgHCF@OHA/HA-ADH/SeMet group exhibited the most homogeneous subchondral trabecular bone structure among all OA groups, closely resembling that of the sham group (Fig. 8D and Fig. S14). Quantitative analysis of subchondral bone parameters further confirmed that intra-articular delivery of these hydrogels effectively reversed OA progression to some extent (Fig. 8E). In summary, the injectable dynamic hydrogels demonstrated excellent properties in maintaining joint structure integrity. When combined with the therapeutic effects of concurrently delivered SeMet and MgHCF, these hydrogels enhance the overall therapeutic efficacy, offering a promising approach for OA treatment.

### Anti-inflammatory evaluation of MgHCF@OHA/HA-ADH/SeMet hydrogels in vivo

**Fig. 9.**
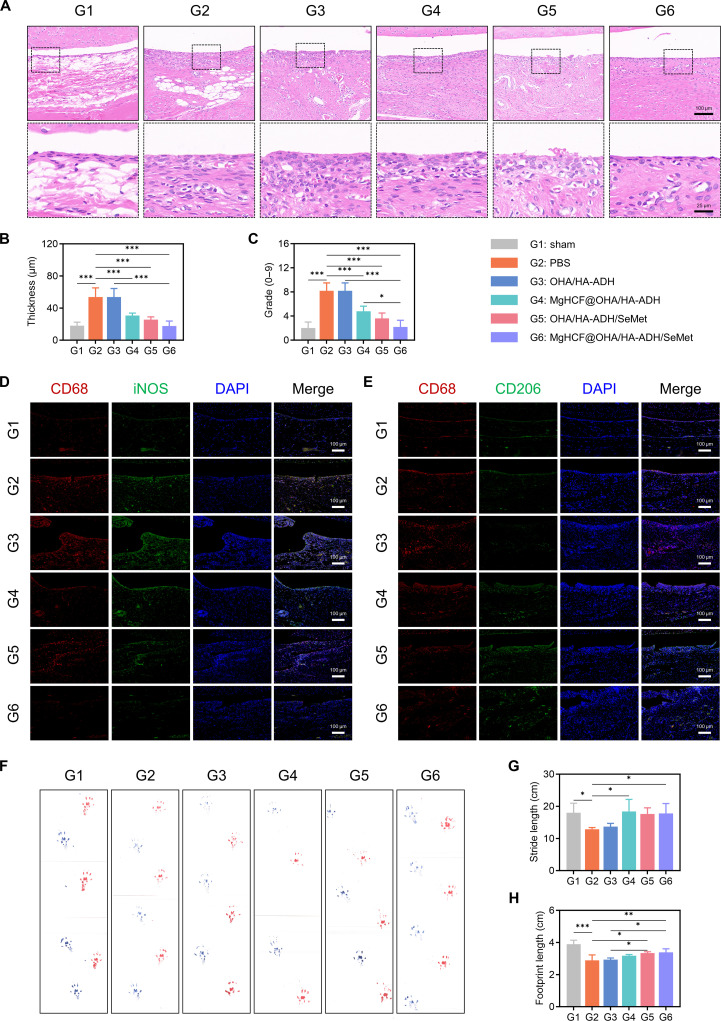
Assessment of inflammatory response and gait tests in vivo. (A) Hematoxylin and eosin (H&E) staining of synovial membranes in MgHCF@OHA/HA-ADH/SeMet-treated joints at 8 weeks. Scale bar, 100 μm. The boxed area is enlarged at the bottom. Scale bar, 25 μm. Quantitative analysis of inflammation in the synovium with H&E, including (B) thickness and (C) grade. Co-immunofluorescence staining of (D) CD68 (red) and inducible nitric oxide synthase (iNOS; green) and (E) CD68 (red) and CD206 (green) in repaired synovial tissues. Scale bar, 100 μm. (F) Footprints of rats 8 weeks after surgery. Red: modeling side; blue: healthy side. Gait assessment for (G) stride length and (H) footprint length. Data are computed as mean ± SD (*n* = 5; “*”, “**”, and “***” denote statistical significance when setting the alpha level to 0.05, 0.01, and 0.001, respectively).

Next, we performed histological evaluation on the morphology, structure, and composition of synovial tissues dissected from the knee joints at week 8 after surgery via hematoxylin and eosin staining. As shown in Fig. 9A, compared with that in the sham group, the synovial lining cell layer in the PBS group was enlarged, with a marked increase in cellularity and infiltration of inflammatory cells, displaying classic pathological features of inflammatory OA. However, the groups treated with other hydrogels exhibited varying degrees of synovial inflammation resolution. Among these, the MgHCF@OHA/HA-ADH/SeMet group demonstrated a notable alleviation of overactivated synovial inflammation, as evidenced by the lowest synovial score and thickness, which were comparable to those of the PBS group (Fig. 9B and C). Based on these in vivo findings, it can be inferred that the intra-articular delivery of MgHCF@OHA/HA-ADH/SeMet hydrogels can ameliorate chronic inflammation. Macrophage infiltration in the synovium plays a critical role in triggering OA-related inflammation [52]. Notably, the immunofluorescence evaluation of synovial macrophage reprogramming showed that the expression of inducible nitric oxide synthase in the MgHCF@OHA/HA-ADH/SeMet group was lower than those in the PBS and OHA/HA-ADH groups (Fig. 9D). Moreover, CD206 expression was increased in the MgHCF@OHA/HA-ADH/SeMet group (Fig. 9E), indicating that these hydrogels could ameliorate OA inflammation-induced synovial macrophage polarization. Inflammatory cytokines released in the synovium can sensitize nociceptive nerve terminals, contributing to inflammatory OA pain. As shown in Fig. 9F, footprints from each experimental group were collected 8 weeks postoperation. Representative footprints, with blue indicating the healthy side and red representing the modeling side, were compared. Subsequent parameter analysis of the collected footprints revealed that the stride length in the hydrogel-treated groups increased to varying degrees, with the MgHCF@OHA/HA-ADH/SeMet group showing a particularly increase compared to the PBS group. Statistical analysis of the footprint length further demonstrated an increase in the MgHCF@OHA/HA-ADH/SeMet group compared to the PBS group (Fig. 9G and H). In summary, these results provide compelling evidence that MgHCF@OHA/HA-ADH/SeMet hydrogels effectively ameliorate inflammation-induced pain in the OA rat model.

### Enhanced cartilage repair effect of MgHCF@OHA/HA-ADH/SeMet hydrogels in vivo

**Fig. 10.**
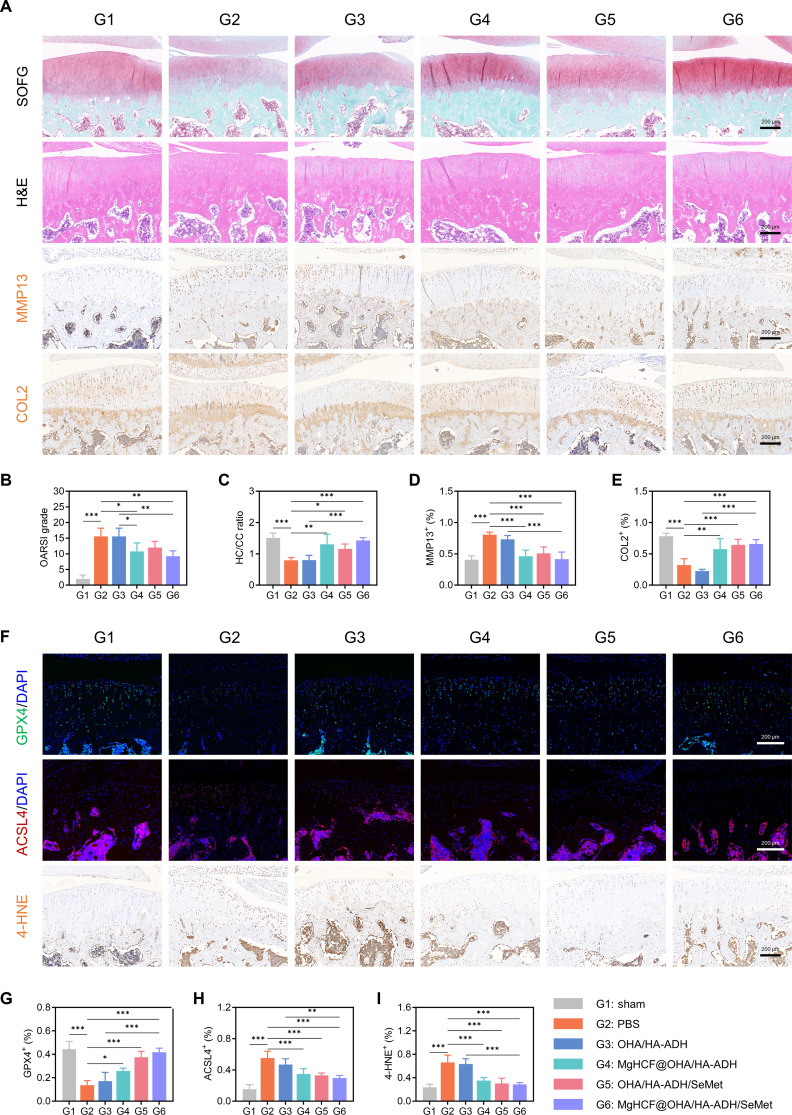
Therapeutic effects of MgHCF@OHA/HA-ADH/SeMet hydrogels on the ECM and ferroptosis. (A) SOFG and H&E staining of joint sections (top 2 rows) and immunohistochemical staining for MMP13 and COL2 of joint sections (bottom 2 rows) at 8 weeks. Scale bar, 200 μm. (B) Medial tibial plateau joint score based on the Osteoarthritis Research Society International (OARSI) scoring system. (C) Ratio of the hyaline cartilage relative to the calcified cartilage. Quantification of (D) MMP13- and (E) COL2-positive chondrocytes in cartilage. (F) Immunofluorescence staining for GPX4 (green) and ACSL4 (red) in cartilage (top 2 rows) and immunohistochemical staining for 4-hydroxynonenal (4-HNE) in cartilage (bottom row) at 8 weeks. Scale bar, 200 μm. (G to I) Quantification of GPX4-, ACSL4-, and 4-HNE-positive chondrocytes in cartilage. Data are computed as mean ± SD (*n* = 5; “*”, “**”, and “***” denote statistical significance when setting the alpha level to 0.05, 0.01, and 0.001, respectively). HC/CC, ratio of the hyaline cartilage relative to the calcified cartilage.

Subsequently, we used Safranin O–Fast Green and hematoxylin and eosin staining to assess histological changes in the knee cartilage at 8 weeks postoperatively. The Safranin O–Fast Green staining results revealed disorganized chondrocytes and a complete disruption of tidemark integrity in the PBS group. In contrast, the MgHCF@OHA/HA-ADH/SeMet group exhibited the highest proteoglycan content, which was comparable to that of the PBS group (Fig. 10A). The above observations were consistent with the Osteoarthritis Research Society International scores and the ratio of hyaline cartilage to calcified cartilage (Fig. 10B and C). To further elucidate the molecular mechanisms underlying the dynamic hydrogel-mediated cartilage regeneration, we evaluated the in vivo expression levels of protein markers associated with ECM metabolism. Immunohistochemical staining for MMP13 demonstrated that treatment with the dynamic hydrogel reduced MMP13 expression (Fig. 10A). Quantitative analysis revealed that the percentage of MMP13-positive cells in the MgHCF@OHA/HA-ADH/SeMet group was comparable to that observed in the sham group (Fig. 10D). In addition, COL2-positive cells were barely detected in the PBS group. However, following the injection of dynamic hydrogels, the number of COL2-positive cells in the cartilage of the treated groups increased progressively (Fig. 10A). Quantitative analysis further indicated that the percentage of COL2-positive cells in the MgHCF@OHA/HA-ADH/SeMet group was comparable to that in the sham group (Fig. 10E). These results suggest that the MgHCF@OHA/HA-ADH/SeMet hydrogels could effectively restore the metabolic balance of the ECM, thereby delaying the progression of OA.

Based on in vitro evidence that MgHCF@OHA/HA-ADH/SeMet hydrogels reduce lipid peroxidation, we further investigated the inhibitory effects of these hydrogels on OA progression through an antiferroptosis mechanism in OA rats. Following treatment with the dynamic hydrogels, we observed an increase in GPX4-positive cells within the cartilage across all hydrogel-treated groups. Quantitative analysis revealed that the percentage of GPX4-positive cells in the MgHCF@OHA/HA-ADH/SeMet group exhibited the most pronounced recovery (Fig. 10F and G). In contrast, compared to that in the sham group, the expression levels of ACSL4 were markedly elevated in the PBS group. However, in the hydrogel-treated groups, ACSL4 expression was reduced to varying degrees. Specifically, quantitative analysis revealed that the percentage of ACSL4-positive cells in the MgHCF@OHA/HA-ADH/SeMet group was comparable to that observed in the sham group (Fig. 10F and H). Next, we evaluated the expression of 4-hydroxynonenal, an advanced lipid peroxidation end product [53], through immunohistochemical staining. The quantitative analysis of 4-hydroxynonenal staining exhibited a consistent trend with the ACSL4 results, further supporting the antiferroptosis effects of the hydrogels (Fig. 10F and I). Moreover, in vivo studies demonstrated the excellent biocompatibility of the MgHCF@OHA/HA-ADH/SeMet hydrogels following local injection (Fig. S15). Overall, these findings indicate that the MgHCF@OHA/HA-ADH/SeMet hydrogels exhibit an optimal ferroptosis inhibition effect, achieved through the synergistic action of MgHCF and SeMet, thereby effectively retarding the process of OA.

## Discussion

OA is the result of interactions of multiple factors, including traumatic injury, metabolic disorders, inflammatory responses, and biochemical changes. The metabolism of trace elements maintains the structure and function of bones and cartilage by participating in processes such as mineralization, matrix synthesis, antioxidation, and anti-inflammation [54]. Notably, additional research is required to completely discover the contents of various trace elements in cartilage, bone, blood, and other tissues, as well as the values of their imbalanced contents and the mechanisms underlying the onset of OA.

Few studies have explored therapeutic strategies capable of regulating multiple trace elements during repair, despite growing evidence that trace elements play a critical role in cartilage regeneration [23]. Our previous study confirmed that IL-1β treatment decreases the expression of selenoproteins in chondrocytes, and administration of hydrogels loaded with selenium nanoparticles induces selenoprotein expression and reduces the ROS level, highlighting that selenium imbalance serves as targets for OA treatment [13]. Organic forms of selenium, principally SeMet, can be directly incorporated into selenoprotein GPXs [25]. Further, SeMet was reported to be covalently immobilized within ultraviolet-responsive gelatin methacryloyl hydrogels via acrylate-functionalized polyethylene glycol tethers, with good plasticity, release properties, and mechanical properties [42]. These consistent conclusions in this study reflect the multiple efficiencies of SeMet. In addition, we uncovered that the total iron ion levels were higher in damaged cartilage than in undamaged cartilage, and iron deposition was detectable in the damaged cartilage. Hydrophilic iron chelators, such as deferoxamine, have demonstrated powerful capabilities in removing intracellular reactive iron [55], but concerns regarding the safety, including hypersensitivity reactions, increased infection risk, and neurotoxicity, remain controversial [56]. The hydrophobicity and poor solubility of curcumin in physiological environments result in low bioavailability despite the highly conjugated β-diketone group in its chemical structure conferring metal-ion-chelating properties [57,58]. Wang et al. [40] designed a dynamic hydrogel with abundant Schiff base moieties as molecule traps to scavenge excessive ferrous ions through coordination. Nevertheless, their chelation effects for ferrous ions are not strong enough compared to chemical capture into hexacyanoferrate nanoparticles [43]. Intra-articular injections of Mg^2+^ at 0.5 mol/l attenuated the progression of OA in rats [59], and the protective effect of Mg^2+^ on OA cartilage was unveiled, primarily mediated by the PI3K/Akt pathway [60]. Thus, the release of Mg^2+^ from the nanoparticles could contribute positively to OA treatment [9].

Highly oxidizable PUFAs promote lipid peroxidation with phospholipids, driving ferroptosis. In our previous work, the AMPK/ACC/ACSL4 axis was shown to reshape PUFA availability and regulate chondrocyte ferroptosis sensitivity [61]. Herein, transcriptomics analysis also highlighted lipid metabolism, indicating that MgHCF@OHA/HA-ADH/SeMet modulated lipid metabolic processes, with the most pronounced effect being ACSL4 down-regulation. To further explore the mechanisms, metabolomics analysis revealed that MgHCF@OHA/HA-ADH/SeMet regulated not only glycerophospholipid metabolism but also cysteine and methionine metabolism. Moreover, MgHCF@OHA/HA-ADH/SeMet increased cysteine metabolism products, including GSH, a key endogenous antioxidant essential for ferroptosis regulation. As an essential component of GSH biosynthesis, cysteine availability is rate limiting, with methionine serving as a metabolic precursor through the transsulfuration pathway [47]. Notably, researchers have demonstrated that augmenting cellular cysteine uptake can effectively scavenge ROS generated via the Fenton reaction, thereby maintaining redox homeostasis [62,63]. SeMet, released from the hydrogel system, may be one of the potential mechanisms by which MgHCF@OHA/HA-ADH/SeMet hydrogels exert their effects. In summary, our multiomics analysis indicates that MgHCF@OHA/HA-ADH/SeMet hydrogels regulate ferroptosis through complex mechanisms, including transcriptional regulation of lipid-metabolism-related genes and metabolic regulation of cysteine and other energy metabolism pathways. Further exploration is needed in the future to fully elucidate these mechanisms.

These results strongly support the potential of the MgHCF@OHA/HA-ADH/SeMet hydrogel in mitigating ferroptosis-driven OA, presenting a promising avenue for the development of innovative therapeutic strategies. Nevertheless, this study still has some limitations. First, using an iron-overloaded or selenium-deficient culture medium as a positive control would support our conclusion. Second, it has been demonstrated that diverse macrophage subsets seeding the synovial joint tissue are implicated in OA pathology. The effect of the MgHCF@OHA/HA-ADH/SeMet hydrogel on macrophage subset polarization needs to be further investigated. With a single administration, the hydrogel, offering both lubrication and anti-inflammation as a localized modulation platform, demonstrates the potential to repair cartilage in OA. MgHCF achieved “attack” on ferroptosis inducers by efficiently capturing Fe^2+^ and sustainedly releasing Mg^2+^, contributing positively to cartilage matrix synthesis; SeMet realized “defense” against oxidative damage by activating GPX4 and scavenging lipid peroxides. However, to facilitate the successful translation of this promising hydrogel from the laboratory to clinical practice, scalable and stable manufacturing processes, detailed cost analyses, ease of use, and patient education should be considered in further study.

## Conclusion

In summary, this study uncovered ferroptosis-related molecular patterns characterized by dysregulated iron metabolism and resistance to lipid peroxidation in degenerative cartilage chondrocytes under OA conditions. Drawing inspiration from the pathogenesis of OA cartilage degeneration, we developed an injectable, dynamically cross-linked HA/SeMet hydrogel integrated with nanoparticles. This construct exhibited robust ferrous ion capture and anti-lipid peroxidation capabilities, facilitating the restoration of ECM metabolic homeostasis. Our findings strongly support the potential of trace element modulated hydrogels to mitigate ferroptosis and promote cartilage matrix reconstruction, presenting a promising avenue for the development of innovative therapeutic strategies.

## Methods

### Synthesis of MgHCF nanoparticles

A mixture of 163 mg of MgCl_2_·6H_2_O and 300 mg of PVP (Mw = 10,000) was dissolved in 40 ml of HCl solution (pH = 1) and stirred for 10 min at room temperature. Subsequently, 40 ml of HCl solution (pH = 1) containing 0.8 mmol of potassium ferricyanide was added dropwise at 80 ml/h. The mixture was stirred for 2 h at room temperature and dialyzed against deionized water for 6 h. The resulting dispersion was freeze-dried to obtain MgHCF nanoparticle powders.

### Preparation of hydrogels

The hydrogel was prepared with equal volumes of 2% (w/w) OHA and 2% (w/w) HA-ADH at room temperature, which was designated as OHA/HA-ADH. Similarly, MgHCF, SeMet, and SeMet + MgHCF were added into the above mixture, thus forming MgHCF@OHA/HA-ADH, OHA/HA-ADH/SeMet, and MgHCF@OHA/HA-ADH/SeMet hydrogels, respectively. The final concentrations of MgHCF and SeMet in the hydrogels were 80 μg/ml and 30 μM, respectively.

### Release kinetics test of hydrogels

One milliliter of MgHCF@OHA/HA-ADH/SeMet hydrogel was immersed in 10 ml of PBS at 37 °C. At predetermined time intervals, 1 ml of the medium was collected and replenished with fresh buffer to restore the initial volume. The concentrations of Se and Mg in the release buffer were quantified using inductively coupled plasma mass spectrometry after digestion with HNO_3_.

### Transcriptional profiling and bioinformatics analysis

The human OA dataset GSE75181 was obtained from the National Center for Biotechnology Information Gene Expression Omnibus database (https://www.ncbi.nlm.nih.gov/geo/). DEGs were identified using the DESeq2 package with adjusted *P* < 0.05 and |log_2_FC| ≥ 1. Functional enrichment of DEGs was assessed via KEGG and GO analyses. Overlapping DEGs with FRGs from the FerrDb database (http://www.zhounan.org/ferrdb/current/) were utilized to construct a protein–protein interaction network through the STRING database (https://string-db.org/). The network was imported into Cytoscape (v3.9.1) and analyzed with the cytoHubba plug-in to identify the top hub genes. Additionally, RNA sequencing of C28/I2 cells was performed by Biotree Biotech Co., Ltd. (Shanghai, China) following standard protocols. DEGs were identified via the Limma package and subjected to bioinformatics analysis.

### Iron ion detection in cartilage

The iron ion levels in cartilage were measured via the Iron Assay Kit (Servicebio, Wuhan, China) as per the manufacturer’s protocol; 100 mg of fresh human OA cartilage tissue was added to 1 ml of assay buffer in a sterile 2-ml tube, and homogenization was conducted on ice at 4 °C. The homogenate was spun at 16,000 × g for 10 min, followed by collection of the supernatant. A detection probe was added to quantify the total iron, followed by incubation at 37 °C for 40 min. Absorbance was assessed at 593 nm via a microplate reader, and total iron levels were assessed for both OA cartilage and corresponding nonlesion cartilage.

### Culture and stimulation of human chondrocytic C28/I2 cells

The immortalized human chondrocyte cell line C28/I2 was acquired from the Cell Bank of Type Culture Collection, Chinese Academy of Sciences. Cells were cultured in Dulbecco’s modified Eagle medium/F-12 medium (Gibco) supplemented with 10% fetal bovine serum, 100 U/ml penicillin, and 100 μg/ml streptomycin (Gibco) in a humidified incubator at 37 °C with 5% CO_2_. The medium was substituted every 3 d. For experiments, C28/I2 cells were exposed to 2 or 5 ng/ml IL-1β (PeproTech, Rocky Hill, NJ) and cultured in hydrogel leachate.

### Lipid peroxidation assay

C28/I2 cells were incubated with 5 μM BODIPY 581/591 C11 (Thermo Fisher, D3861) for 40 min, rinsed with PBS, trypsinized, and filtered to obtain single-cell suspensions. Flow cytometry was conducted via BD FACS Aria II (Becton Dickinson). Upon oxidation, the excitation maximum shifted from 581 to 500 nm, and the emission maximum, from 591 to 510 nm. Oxidized BODIPY-C11 was detected with a fluorescein isothiocyanate filter (emission 510 nm). Data were analyzed by microscopy or FlowJo v10 (BD Biosciences).

### Detection of the mitochondrial network and membrane potential

For mitochondrial staining, the incubation of cells was conducted with MitoTracker Red (Beyotime, China) at 37 °C for 30 min, followed by washing with PBS. To assess mitochondrial membrane potential, cells were analyzed using the JC-1 Mitochondrial Membrane Potential Assay Kit (Beyotime, China). Briefly, the incubation of cells was conducted with 1× JC-1 staining solution for 20 min in the dark at 37 °C. Confocal microscopy photographs were taken using either a Zeiss LSM 710 laser scanning microscope or a Leica TCS SP8 STED confocal microscope.

### Gait analysis

To evaluate alterations in the gait of rats, gait analysis was conducted. The right feet of the rats were marked with red ink, while the left feet were marked with blue ink. The rats were then led to walk naturally across a sheet of paper to obtain their footprints. These footprints were manually analyzed, and specific parameters, such as stride length and print area, were quantified. Notably, the first few footprints were typically excluded from statistical analysis, as the rats might not yet be walking consistently during this initial period.

### Micro-CT analysis

The knee joint specimens were initially fixed in 4% paraformaldehyde for 48 h, 3 washes were conducted with PBS, and the specimens were subsequently stored in 70% ethanol. Scanning and reconstruction of the samples were conducted using high-resolution micro-CT (SkyScan 1172) and CT reconstruction software (NRecon v1.6). X-ray photographs were acquired to assess the joint space widths. For 3-dimensional model visualization and further data analysis, CTAn v1.9 and CTVol v2.0 were utilized. Micro-CT scanning was performed with the following parameters: x-ray voltage set at 50 kV, source current at 200 μA, and a resolution of 10 μm. Following the scanning process, 3-dimensional reconstruction was carried out, and the subchondral bone parameters were analyzed under consistent conditions.

### Statistical analysis

The analysis was performed in a blinded way. GraphPad Prism 9.0 was utilized to conduct statistical analyses (GraphPad Software, La Jolla, USA). For comparisons between 2 groups, 2-tailed unpaired *t* tests were used, while one-way analysis of variance followed by Tukey–Kramer post hoc tests was applied for comparisons among 3 or more groups. Data are reported as mean ± standard deviation, with *P* < 0.05 regarded as significance.

## Data Availability

Data are available upon reasonable request.

## References

[1] Butterfield DA, Halliwell B. Oxidative stress, dysfunctional glucose metabolism and Alzheimer disease. Nat Rev Neurosci. 2019;20(3):148–160. 30737462 10.1038/s41583-019-0132-6 PMC9382875

[2] Andersen JK. Oxidative stress in neurodegeneration: Cause or consequence? Nat Med. 2004;10(Suppl):S18–S25. 15298006 10.1038/nrn1434

[3] Liu Y, Nguyen M, Robert A, Meunier B. Metal ions in Alzheimer’s disease: A key role or not? Acc Chem Res. 2019;52(7):2026–2035. 31274278 10.1021/acs.accounts.9b00248

[4] Xiao C, Li J, Hua A, Wang X, Li S, Li Z, Xu C, Zhang Z, Yang X, Li Z. Hyperbaric oxygen boosts antitumor efficacy of copper-diethyldithiocarbamate nanoparticles against pancreatic ductal adenocarcinoma by regulating cancer stem cell metabolism. Research. 2024;7:0335. 38766644 10.34133/research.0335 PMC11100349

[5] Zhang J, Hu W, Li Y, Kang F, Yao X, Li J, Dong S. MAGI1 attenuates osteoarthritis by regulating osteoclast fusion in subchondral bone through the RhoA-ROCK1 signaling pathway. J Orthop Translat. 2025;52:167–181. 40322041 10.1016/j.jot.2025.04.007 PMC12049846

[6] Katz JN, Arant KR, Loeser RF. Diagnosis and treatment of hip and knee osteoarthritis: A review. JAMA. 2021;325(6):568–578. 33560326 10.1001/jama.2020.22171 PMC8225295

[7] Yao Q, Wu X, Tao C, Gong W, Chen M, Qu M, Zhong Y, He T, Chen S, Xiao G. Osteoarthritis: Pathogenic signaling pathways and therapeutic targets. Signal Transduct Target Ther. 2023;8(1):56. 36737426 10.1038/s41392-023-01330-w PMC9898571

[8] Yang WM, Lv JF, Wang YY, Xu YM, Lin J, Liu J, Chen JJ, Wang XZ. The daily intake levels of copper, selenium, and zinc are associated with osteoarthritis but not with rheumatoid arthritis in a cross-sectional study. Biol Trace Elem Res. 2023;201(12):5662–5670. 36943549 10.1007/s12011-023-03636-w

[9] Kuang X, Chiou J, Lo K, Wen C. Magnesium in joint health and osteoarthritis. Nutr Res. 2021;90:24–35. 34023805 10.1016/j.nutres.2021.03.002

[10] Chang B, Hu Z, Chen L, Jin Z, Yang Y. Development and validation of cuproptosis-related genes in synovitis during osteoarthritis progress. Front Immunol. 2023;14:1090596. 36817415 10.3389/fimmu.2023.1090596 PMC9932029

[11] Ru Q, Li Y, Xie W, Ding Y, Chen L, Xu G, Wu Y, Wang F. Fighting age-related orthopedic diseases: Focusing on ferroptosis. Bone Res. 2023;11(1):12. 36854703 10.1038/s41413-023-00247-y PMC9975200

[12] Cai C, Hu W, Chu T. Interplay between iron overload and osteoarthritis: Clinical significance and cellular mechanisms. Front Cell Dev Biol. 2021;9: Article 817104. 35096841 10.3389/fcell.2021.817104 PMC8795893

[13] Hu W, Yao X, Li Y, Li J, Zhang J, Zou Z, Kang F, Dong S. Injectable hydrogel with selenium nanoparticles delivery for sustained glutathione peroxidase activation and enhanced osteoarthritis therapeutics. Mater Today Bio. 2023;23: Article 100864. 10.1016/j.mtbio.2023.100864 PMC10679772 38024839

[14] Zhong C, Yang J, Zhang Y, Fan X, Fan Y, Hua N, Li D, Jin S, Li Y, Chen P, et al. TRPM2 mediates hepatic ischemia–reperfusion injury via Ca^2+^-induced mitochondrial lipid peroxidation through increasing ALOX12 expression. Research. 2023;6:0159. 37275121 10.34133/research.0159 PMC10232356

[15] Guo Z, Lin J, Sun K, Guo J, Yao X, Wang G, Hou L, Xu J, Guo J, Guo F. Deferoxamine alleviates osteoarthritis by inhibiting chondrocyte ferroptosis and activating the Nrf2 pathway. Front Pharmacol. 2022;13: Article 791376. 35359876 10.3389/fphar.2022.791376 PMC8964096

[16] Kang D, Lee J, Jung J, Carlson BA, Chang MJ, Chang CB, Kang SB, Lee BC, Gladyshev VN, Hatfield DL, et al. Selenophosphate synthetase 1 deficiency exacerbates osteoarthritis by dysregulating redox homeostasis. Nat Commun. 2022;13(1):779. 35140209 10.1038/s41467-022-28385-7 PMC8828855

[17] Wu W, Yu L, Pu Y, Yao H, Chen Y, Shi J. Copper-enriched Prussian blue nanomedicine for in situ disulfiram toxification and photothermal antitumor amplification. Adv Mater. 2020;32(17): Article e2000542. 32162734 10.1002/adma.202000542

[18] Chen Y, Li ZH, Pan P, Hu JJ, Cheng SX, Zhang XZ. Tumor-microenvironment-triggered ion exchange of a metal–organic framework hybrid for multimodal imaging and synergistic therapy of tumors. Adv Mater. 2020;32(24): Article e2001452. 32374492 10.1002/adma.202001452

[19] Zhang W, Hu S, Yin JJ, He W, Lu W, Ma M, Gu N, Zhang Y. Prussian blue nanoparticles as multienzyme mimetics and reactive oxygen species scavengers. J Am Chem Soc. 2016;138(18):5860–5865. 26918394 10.1021/jacs.5b12070

[20] Cai X, Zhang K, Xie X, Zhu X, Feng J, Jin Z, Zhang H, Tian M, Chen H. Self-assembly hollow manganese Prussian white nanocapsules attenuate Tau-related neuropathology and cognitive decline. Biomaterials. 2020;231: Article 119678. 31864019 10.1016/j.biomaterials.2019.119678

[21] Wang Z, Yu B, Alamri H, Yarabarla S, Kim MH, Huang SD. KCa(H_2_O)_2_[Fe^III^(CN)_6_]·H_2_O nanoparticles as an antimicrobial agent against *Staphylococcus aureus*. Angew Chem Int Ed Engl. 2018;57(8):2214–2218. 29392801 10.1002/anie.201713177 PMC6358163

[22] Huang Q, Jiang C, Xia X, Wang Y, Yan C, Wang X, Lei T, Yang X, Yang W, Cheng G, et al. Pathological BBB crossing melanin-like nanoparticles as metal-ion chelators and neuroinflammation regulators against Alzheimer’s disease. Research. 2023;6:0180. 37363131 10.34133/research.0180 PMC10289297

[23] Zhou H, Yao X, Liu S, Li Y, Hu L, Zhang J, Hu W, Dong S. Advances in selenium research for bone and joint-related diseases: From pathophysiological mechanisms to therapeutic implications of selenium-based biomaterials. Biomater Transl. 2025; 10.12336/bmt.25.00002.

[24] Vinceti M, Filippini T, Jablonska E, Saito Y, Wise LA. Safety of selenium exposure and limitations of selenoprotein maximization: Molecular and epidemiologic perspectives. Environ Res. 2022;211: Article 113092. 35259406 10.1016/j.envres.2022.113092

[25] Ingold I, Berndt C, Schmitt S, Doll S, Poschmann G, Buday K, Roveri A, Peng X, Porto Freitas F, Seibt T, et al. Selenium utilization by GPX4 is required to prevent hydroperoxide-induced ferroptosis. Cell. 2018;172(3):409–422.e421. 29290465 10.1016/j.cell.2017.11.048

[26] Huang C, Guo Y, Li T, Sun G, Yang J, Wang Y, Xiang Y, Wang L, Jin M, Li J, et al. Pharmacological activation of GPX4 ameliorates doxorubicin-induced cardiomyopathy. Redox Biol. 2024;70: Article 103024. 38232458 10.1016/j.redox.2023.103024 PMC10827549

[27] Makarević J, Jokić M, Frkanec L, Katalenić D, Zinić M. Gels with exceptional thermal stability formed by bis(amino acid) oxalamide gelators and solvents of low polarity. Chem Commun (Camb). 2002;19:2238–2239. 10.1039/b206690d 12397995

[28] Li G, Shi Z, Zong H, Zhang K, Yan S, Yin J. Injectable, self-healing poly(amino acid)-hydrogel based on phenylboronate ester bond for osteochondral tissue engineering. Biomed Mater. 2023;18(5). 10.1088/1748-605X/ace39b 37399811

[29] Rana MM, De la Hoz Siegler H. Evolution of hybrid hydrogels: Next-generation biomaterials for drug delivery and tissue engineering. Gels. 2024;10(4):216. 38667635 10.3390/gels10040216 PMC11049329

[30] Chen W, Zhang H, Zhou Q, Zhou F, Zhang Q, Su J. Smart hydrogels for bone reconstruction via modulating the microenvironment. Research. 2023;6:0089. 36996343 10.34133/research.0089 PMC10042443

[31] Li G, Liu S, Chen Y, Zhao J, Xu H, Weng J, Yu F, Xiong A, Udduttula A, Wang D, et al. An injectable liposome-anchored teriparatide incorporated gallic acid-grafted gelatin hydrogel for osteoarthritis treatment. Nat Commun. 2023;14(1):3159. 37258510 10.1038/s41467-023-38597-0 PMC10232438

[32] Vinikoor T, Dzidotor GK, Le TT, Liu Y, Kan HM, Barui S, Chorsi MT, Curry EJ, Reinhardt E, Wang H, et al. Injectable and biodegradable piezoelectric hydrogel for osteoarthritis treatment. Nat Commun. 2023;14(1):6257. 37802985 10.1038/s41467-023-41594-y PMC10558537

[33] Chung C, Burdick JA. Influence of three-dimensional hyaluronic acid microenvironments on mesenchymal stem cell chondrogenesis. Tissue Eng Part A. 2009;15(2):243–254. 19193129 10.1089/ten.tea.2008.0067 PMC2678568

[34] Li J, Ma J, Feng Q, Xie E, Meng Q, Shu W, Wu J, Bian L, Han F, Li B. Building osteogenic microenvironments with a double-network composite hydrogel for bone repair. Research. 2023;6:0021. 37040486 10.34133/research.0021 PMC10076009

[35] Song X, He S, Zheng J, Yang S, Li Q, Zhang Y. One-step construction of tryptophan-derived small molecule hydrogels for antibacterial materials. Molecules. 2023;28(8):3334. 37110568 10.3390/molecules28083334 PMC10141015

[36] Luo X, Yuan Z, Xie X, Xie Y, Lv H, Zhao J, Wang H, Gao Y, Zhao L, Wang Y, et al. Amino acid-induced rapid gelation and mechanical reinforcement of hydrogels with low-hysteresis and self-recoverable and fatigue-resistant properties. Mater Horiz. 2023;10(10):4303–4316. 37697907 10.1039/d3mh00483j

[37] Liu Y, Wan Y, Jiang Y, Zhang L, Cheng W. GPX4: The hub of lipid oxidation, ferroptosis, disease and treatment. Biochim Biophys Acta Rev Cancer. 2023;1878(3): Article 188890. 37001616 10.1016/j.bbcan.2023.188890

[38] Hou J, Jiang C, Wen X, Li C, Xiong S, Yue T, Long P, Shi J, Zhang Z. ACSL4 as a potential target and biomarker for anticancer: From molecular mechanisms to clinical therapeutics. Front Pharmacol. 2022;13: Article 949863. 35910359 10.3389/fphar.2022.949863 PMC9326356

[39] Pei Z, Qin Y, Fu X, Yang F, Huo F, Liang X, Wang S, Cui H, Lin P, Zhou G, et al. Inhibition of ferroptosis and iron accumulation alleviates pulmonary fibrosis in a bleomycin model. Redox Biol. 2022;57: Article 102509. 36302319 10.1016/j.redox.2022.102509 PMC9614651

[40] Wang Y, Tan L, Zhang W, Yang Y, Li C, Li H, Cai K, Hu Y, Luo Z, Liu M. Injectable dynamic hydrogel with responsive mechanical reinforcing ability reverses intervertebral disc degeneration by suppressing ferroptosis and restoring matrix homeostasis. Adv Funct Mater. 2024;34(14):2310416.

[41] Yu H, Zhou C, Yang S, Yu J, Zhang X, Liang Z, Tan S, Song Y, Wang W, Sun Y, et al. Mitigation of arteriosclerosis through transcriptional regulation of ferroptosis and lipid metabolism by magnesium. Biomaterials. 2025;319: Article 123135. 39985976 10.1016/j.biomaterials.2025.123135

[42] Sun J, Xie X, Song Y, Sun T, Liu X, Yuan H, Shen C. Selenomethionine in gelatin methacryloyl hydrogels: Modulating ferroptosis to attenuate skin aging. Bioact Mater. 2024;35:495–516. 38404642 10.1016/j.bioactmat.2024.02.013 PMC10885793

[43] Huo M, Tang Z, Wang L, Zhang L, Guo H, Chen Y, Gu P, Shi J. Magnesium hexacyanoferrate nanocatalysts attenuate chemodrug-induced cardiotoxicity through an anti-apoptosis mechanism driven by modulation of ferrous iron. Nat Commun. 2022;13(1):7778. 36522337 10.1038/s41467-022-35503-y PMC9755285

[44] Luo M, Hong Y, Yao W, Huang C, Xu Q, Wu Q. Facile removal of polyvinylpyrrolidone (PVP) adsorbates from Pt alloy nanoparticles. J Mater Chem A. 2015;3(6):2770–2775.

[45] Abramoff B, Caldera FE. Osteoarthritis: Pathology, diagnosis, and treatment options. Med Clin North Am. 2020;104(2):293–311. 32035570 10.1016/j.mcna.2019.10.007

[46] Gorospe CM, Carvalho G, Herrera Curbelo A, Marchhart L, Mendes IC, Niedźwiecka K, Wanrooij PH. Mitochondrial membrane potential acts as a retrograde signal to regulate cell cycle progression. Life Sci Alliance. 2023;6(12): Article e202302091. 37696576 10.26508/lsa.202302091 PMC10494934

[47] Jiang X, Stockwell BR, Conrad M. Ferroptosis: Mechanisms, biology and role in disease. Nat Rev Mol Cell Biol. 2021;22(4):266–282. 33495651 10.1038/s41580-020-00324-8 PMC8142022

[48] Zhou X, Pan Y, Li J, Zhuang R, Tong P, Xia H. Notopterol mitigates osteoarthritis progression and relieves pain in mice by inhibiting PI3K/Akt/GPX4-mediated ferroptosis. Int Immunopharmacol. 2025;151: Article 114323. 40020461 10.1016/j.intimp.2025.114323

[49] Ioannilli L, Ciccarone F, Ciriolo MR. Adipose tissue and FoxO1: Bridging physiology and mechanisms. Cells. 2020;9(4):849. 32244542 10.3390/cells9040849 PMC7226803

[50] Wang X, Ji Y, Qi J, Zhou S, Wan S, Fan C, Gu Z, An P, Luo Y, Luo J. *Mitochondrial carrier 1* (*MTCH1*) governs ferroptosis by triggering the FoxO1-GPX4 axis-mediated retrograde signaling in cervical cancer cells. Cell Death Dis. 2023;14(8):508. 37550282 10.1038/s41419-023-06033-2 PMC10406804

[51] Upadhyayula PS, Higgins DM, Mela A, Banu M, Dovas A, Zandkarimi F, Patel P, Mahajan A, Humala N, Nguyen TTT, et al. Dietary restriction of cysteine and methionine sensitizes gliomas to ferroptosis and induces alterations in energetic metabolism. Nat Commun. 2023;14(1):1187. 36864031 10.1038/s41467-023-36630-w PMC9981683

[52] Miyahara J, Omata Y, Chijimatsu R, Okada H, Ishikura H, Higuchi J, Tachibana N, Nagata K, Tani S, Kono K, et al. *CD34^hi^* subset of synovial fibroblasts contributes to fibrotic phenotype of human knee osteoarthritis. JCI Insight. 2025;10(2): Article e183690. 39846253 10.1172/jci.insight.183690 PMC11790023

[53] Li Pomi F, Gammeri L, Borgia F, Di Gioacchino M, Gangemi S. Oxidative stress and skin diseases: The role of lipid peroxidation. Antioxidants. 2025;14(5):555. 40427437 10.3390/antiox14050555 PMC12108378

[54] Amhare AF, Liu H, Qiao L, Deng H, Han J. Elemental influence: The emerging role of zinc, copper, and selenium in osteoarthritis. Nutrients. 2025;17(13):2069. 40647177 10.3390/nu17132069 PMC12251344

[55] Feng W, Xiao Y, Zhao C, Zhang Z, Liu W, Ma J, Ganz T, Zhang J, Liu S. New deferric amine compounds efficiently chelate excess iron to treat iron overload disorders and to prevent ferroptosis. Adv Sci. 2022;9(29): Article e2202679. 10.1002/advs.202202679 PMC9561787 36031399

[56] Kang H, Han M, Xue J, Baek Y, Chang J, Hu S, Nam H, Jo MJ, El Fakhri G, Hutchens MP, et al. Renal clearable nanochelators for iron overload therapy. Nat Commun. 2019;10(1):5134. 31723130 10.1038/s41467-019-13143-z PMC6853917

[57] Wanninger S, Lorenz V, Subhan A, Edelmann FT. Metal complexes of curcumin—Synthetic strategies, structures and medicinal applications. Chem Soc Rev. 2015;44(15):4986–5002. 25964104 10.1039/c5cs00088b

[58] Liu J, Gao D, Hu D, Lan S, Liu Y, Zheng H, Yuan Z, Sheng Z. Delivery of biomimetic liposomes via meningeal lymphatic vessels route for targeted therapy of Parkinson’s disease. Research. 2023;6:0030. 37040500 10.34133/research.0030 PMC10076012

[59] Yao H, Xu JK, Zheng NY, Wang JL, Mok SW, Lee YW, Shi L, Wang JY, Yue J, Yung SH, et al. Intra-articular injection of magnesium chloride attenuates osteoarthritis progression in rats. Osteoart Cartil. 2019;27(12):1811–1821. 10.1016/j.joca.2019.08.007 31536815

[60] Zheng L, Zhao S, Li Y, Xu J, Yan W, Guo B, Xu J, Jiang L, Zhang Y, Wei H, et al. Engineered MgO nanoparticles for cartilage-bone synergistic therapy. Sci Adv. 2024;10(10): Article eadk6084. 38457498 10.1126/sciadv.adk6084 PMC10923500

[61] Zou Z, Hu W, Kang F, Xu Z, Li Y, Zhang J, Li J, Zhang Y, Dong S. Interplay between lipid dysregulation and ferroptosis in chondrocytes and the targeted therapy effect of metformin on osteoarthritis. J Adv Res. 2025;69:515–529. 38621621 10.1016/j.jare.2024.04.012 PMC11954841

[62] Brosnan JT, Brosnan ME. The sulfur-containing amino acids: An overview. J Nutr. 2006;136(6 Suppl):1636s–1640s. 16702333 10.1093/jn/136.6.1636S

[63] Floros KV, Chawla AT, Johnson-Berro MO, Khatri R, Stamatouli AM, Boikos SA, Dozmorov MG, Cowart LA, Faber AC. MYCN upregulates the transsulfuration pathway to suppress the ferroptotic vulnerability in *MYCN*-amplified neuroblastoma. Cell Stress. 2022;6(2):21–29. 35174317 10.15698/cst2022.02.264 PMC8802432

