# Supplementary material for: An Attack and Defense Strategy for Osteoarthritis Repair: Constructing a Trace Element Modulated Hydrogel to Mitigate Ferroptosis and Promote Cartilage Matrix Reconstruction
Source: Research (Wash D C). 2025 Nov 11;8:0979. doi: 10.34133/research.0979 (PMC12604057; doi:10.34133/research.0979)
Supplement: Supplementary 1 — Supplementary Text Figs. S1 to S15 Tables S1 to S3 [file research.0979.f1.docx]

Supplementary Materials for

**An Attack and Defense Strategy for Osteoarthritis Repair: Constructing a Trace Element Modulated Hydrogel to Mitigate Ferroptosis and Promote Cartilage Matrix Reconstruction**

Wenhui Hu^1,2,3^†, Fei Kang^1^†, Yuheng Li^1^†, Yixiang Xu^1^, Xiaoming Li^4^, Jing Zhang^5^,

Jie Liao^1^, Jingjin Dai^1^, Xiaoshan Gong^1^, Jianmei Li^1^*, Xuan Yao^6^*, Shiwu Dong^1,2^*

^1^Department of Biomedical Materials Science, College of Biomedical Engineering,

Third Military Medical University, Chongqing 400038, China.

^2^State Key Laboratory of Trauma and Chemical Poisoning, Third Military Medical University, Chongqing 400038, China.

^3^Department of Basic Medicine, Frontier Medical Service Training Brigade, Third Military Medical University, Changji, Xinjiang 831200, P.R. China.

^4^Department of Military Traffic Medicine, Daping Hospital, Third Military Medical University, Chongqing 400042, China.

^5^College of Bioengineering, Chongqing University, Chongqing 400044, China.

^6^Department of Clinical Hematology Faculty of Laboratory Medicine, Third Military Medical University, Chongqing 400038, China.

*Address correspondence to:

lijianmei@tmmu.edu.cn (J. L);

yao_xuan1129@tmmu.edu.cn (X. Y);

dongshiwu@tmmu.edu.cn (S. D)

†These authors contributed equally to this work.

**This PDF file includes:**

Supplementary Text

Figs. S1 to S15

Tables S1 to S3

**Supplementary Text**

Experimental Section

*Characterization of MgHCF Nanoparticles*: Transmission electron microscope (TEM) was adopted for morphology observation on EOL JEM-F200 electron microscope. Energy dispersive spectrometry (EDS) mapping was performed by dispersing MgHCF nanoparticles onto the substrate, loading them into the EDS-equipped microscope (JEOL JEM-F200), acquiring elemental maps, and analyzing the data to determine elemental distribution. Hydrodynamic diameter was obtained on Malvern Zetasizer Nano ZS90 following the dynamic light scattering (DLS) method. X-ray diffraction (XRD, Rigaku SmartLab SE, Japan) was conducted to identify the MgHCF nanoparticles in the range of 10-70° with a scan rate of 2°/min. Fourier-transform infrared spectroscopy (FTIR) was acquired on Nicolet iS20 spectrometer using KBr pellets. Raman spectra were collected on Horiba LabRAM HR Evolution with a 473 nm laser line.

*Ferrous Binding Assay*: 1 mL of serial concentrations of the aqueous solution containing 40, 20, 10, 5, 2.5, 1.25 and 0 μg/mL ferrous sulfate was added into the aqueous solution of MgHCF nanoparticles (40 μg/mL) in identical volume. The mixture was allowed for 30 s equilibrium. For ferrous binding assays in the dialysis membrane isolated cation exchange process, 10 mL of the MgHCF nanoparticles (1 mg/mL) was sealed into the dialysis bag and immersed into the beaker containing 150 mL solution of ferrous sulfate with stirring. At predetermined time points, aliquots of the solution in the beaker were collected for elemental analysis (Mg, Fe) using ICP-MS.

*Synthesis of OHA and HA-ADH*: OHA was fabricated according to the sodium periodate oxidation method. Briefly, 1 g HA was dissolved in 100 mL distilled water, and 0.3 sodium periodate was added subsequently. The reaction was stirred for 24 h at room temperature. Diethylene glycol was added in an equimolar amount to stop the oxidation reaction for 1 h, and the mixture underwent exhaustive dialysis for 3 days against water. To obtained HA-ADH, 1g of HA was dissolved in 100 mL of MES buffer (pH = 6.5), and EDC (1.25 g) and HOBt (0.89 g) were added sequentially for 1 h. Then, 4.5g ADH was added for another 24 h at room temperature. After the above reaction was complete, the mixture was dialyzed exhaustively for 4 days against water.

*FTIR and NMR:* The successful synthesis of OHA, OHA/SeMet, and HA-ADH was verified using FTIR; ^1^H nuclear magnetic resonance (NMR, Bruker DMX, German) was applied to characterize the chemical structures of samples.

*Release Kinetics Test of Hydrogels*: 1 mL MgHCF@OHA/HA-ADH/SeMet hydrogel was immersed in 10 mL PBS at 37 °C. At predetermined time intervals, 1 mL of the medium was collected and replenished with fresh buffer to restore the initial volume. The concentrations of Se and Mg in the release buffer were quantified using ICP-MS after digestion with HNO_3_.

*Gelation Time, Swelling Assay, and Degradation Behavior*: The gelation time of hydrogel was determined as the time when the sample stopped flowing. To assess the swelling kinetics of the hydrogels, the gelled hydrogels (W_0_) were soaked in PBS solution. After 24 h, the hydrogels were removed from solution and the excess water on its surface was blotted with filter paper. Its weight was recorded as Ww. The swelling ratio (SR) was calculated by the formula: SR= (W_w_-W_0_)/W_0_ × 100%. The degradation behavior of the hydrogels was assessed by wet weight loss. Briefly, 200 μL hydrogel was immersed in 1 mL PBS at 37°C. The residual weight of hydrogel was recorded at scheduled times.

*SEM Observation and Compression Test*: The microstructure of the hydrogels was observed using a scanning electron microscope (SEM) (German, ZEISS Sigma 300). Before observation, the hydrogels were lyophilized and sprayed with gold. The pore sizes of the hydrogels were measured by Image J software according to SEM images. Compression testing was carried out to evaluate the mechanical property by applying a strain rate of 1 mm/min using a universal testing system (CMT6103, USA). Three replicates were used for each group.

*Self-Healing Ability and Injectable Properties Test*: The dynamic rheological experiments of hydrogels were determined using a rheometer (Anton Paar MCR 302, Austria) at room temperature. Hydrogel (1 mL) was placed on a parallel plate (20 mm). The storage modulus (G′) and loss modulus (G′′) were measured by time sweep experiments using a fixed train of 1% and a frequency of 1 Hz. To detect the critical strain point in hydrogels, the strain amplitude sweep test was used at a fixed frequency of 1 Hz with a strain ranging from 0.1% to 1000%. To confirm the self-healing ability of hydrogels, the continuous step-strain test was conducted. In brief, the oscillatory strain was alternated between a small strain of 1% and a large strain of 100% at a fixed frequency of 1 Hz, keeping a duration of 120 s for each step. To observe the injectability of the MgHCF@OHA/HA-ADH/SeMet hydrogel, the hydrogel was placed in a syringe with a 26-gauge needle and then injected through a needle directly on a plate. Three pieces of hydrogels dyed white, pink and blue were put together and merged into a single integral hydrogel again without any external stimulus to further illustrate its self-healing performance.

*Patients and Specimens*: All human studies were conducted with informed consent of the patients and approval of the Institutional Ethics Review Board of the Second Affiliated Hospital of Third Military Medical University (Approval number: 2023-yandi056-01). OA was macroscopically diagnosed according to the Modified Outerbridge Classification. Articular cartilage samples were collected from patients with knee OA who underwent knee arthroplasty surgery. Specimens that included all cartilage layers and subchondral bone were separately harvested from sites on the tibial plateau by drilling holes. Clinical information was collected from patient records.

*Enhanced Perls’ Prussian Blue (EPPB) Staining*: An Iron Stain kit was used to identify iron pigments (AR15811-2, Artisan, Dako, Agilent). Staining was performed in a Dako ArtisanLink (Dako, Agilent) staining system according to the manufacturer’s instructions. To enhance iron detection, we adapted the EPPB staining protocol [1] by adding a blocking step with 5% normal goat serum (16210064, Life Technology) with 2.5% BSA (10735078001, Sigma) for 60 min followed with 30 min of peroxidase-blocking solution (S2023, Dako-Agilent) and a 30 min incubation with Liquid DAB+ Substrate Chromogen System (K3468, Dako-Agilent) on sections that had been previously stained with the Iron Stain kit.

*Determination of Intracellular Ferrous Iron (Fe^2+^) and GSH Levels*: Intracellular ferrous iron level was determined using the iron assay kit purchased from Abcam and was used according to the manufacturer’s instructions. Briefly, samples were collected and washed in cold PBS. Samples were homogenized in 5X volumes of iron assay buffer on ice. The supernatant was collected and iron reducer was added to each sample before mixing, and incubating for 30 min. Then, the iron probe was added to each sample before mixing, and incubating for 60 min. The output was measured immediately on a colorimetric microplate reader (optical density (OD) 593 nm). In addition, the intracellular ferrous ion was evaluated with FerroOrange (F374, Dojindo, Japan) according to their manufacturer’s instructions. The acquisition of confocal microscopic images was performed using either a Zeiss laser scanning microscope 710 or a Leica TCS SP8 STED confocal microscope. The intracellular levels of GSH (S0053) were determined using the corresponding kits from Beyotime. The experiments were conducted according to the manufacturers’ instructions.

*Quantitative Real-time Polymerase Chain Reaction (qRT-PCR)*: RNA extraction was performed on C28/I2 chondrocytes. Employing TRIzol reagent (Takara) in line to the manufacturer's instructions. The quantification of extracted total RNA was performed employing a NanoDrop spectrophotometer (Thermo Fisher Scientific, Wilmington, DE, USA). Following that, a 2-µg amount of total RNA was employed for reverse transcription to produce complementary DNA (cDNA) utilizing the Prime Script RT Reagent Kit (Takara), following the manufacturer's recommendations. The qRT-PCR analysis was performed employing gene-specific primers and SYBR Green (Invitrogen) as the fluorescent probe. The analysis was carried out on a 7900HT Fast Time PCR machine. The primer sequences are provided in Table S1.

*Immunofluorescence*: Briefly, C28/I2 cells were incubated with immunostaining permeabilization buffer with Triton X-100 (Beyotime, China) for 10 min to facilitate antigen exposure. Then the samples were subjected to blocking buffer before incubation with primary antibodies against COL2 (1:200, Bioss, bs-10589R), MMP13 (1:300, Bioworld, BS79990), ACSL4 (1:200, Affinity, DF12141), GPX4 (1:200, Afﬁnity, DF6701), and FoxO1 (1:200, Bioss, bs-2537R) at 4 °C overnight, respectively. After being washed with PBS, the samples were incubated with secondary Alexa 488-or Alexa 647-labeled antibody at 37 °C for 1 h. The acquisition of confocal microscopic images was performed using either a Zeiss laser scanning microscope 710 or a Leica TCS SP8 STED confocal microscope. The antibodies used in this study are shown in Table S2.

*Cytocompatibility Evaluation*: For the CCK-8 assay, the medium in the well was extracted and substituted with 100 μL of DMEM/F-12 solution containing 10% CCK-8 dye at the determined time point. The solution was incubated for 1 h at a temperature of 37 °C, and the absorbance was then measured at a wavelength of 450 nanometers using a microplate reader. For the Live/Dead assay, 100 μL of Calcein-AM/PI dye was used instead of the CCK-8 dye. Following incubation at a temperature of 37 °C for a duration of 25-30 minutes, the cells were examined and captured using an inverted fluorescent microscope (Bio-Rad, USA).

*Transmission Electron Microscopy (TEM)*: C28/I2 chondrocytes treated with MgHCF@OHA/HA-ADH/SeMet hydrogels were fixed with 1% formaldehyde and 2.5% glutaraldehyde in 0.1 M Sodium cacodylate buffer for 1 h at room temperature, washed 3 times in cacodylate buffer, then post-fixed in 1% osmium tetroxide in H_2_O for 1 h and washed in H_2_O for 3 times. Next, samples were dehydrated in graded ethanol concentrations and propylene oxide, and embedded in Spurr’s resin. Ultrathin frozen sections were then prepared and transferred to copper grids, stained with 0.75% uranyl acetate or 1% lead citrate, and observed by TEM (HT7700, Hitachi, Japan).

*Western Blot Analysis*: The cells were rinsed twice with ice-cold PBS, lysed with RIPA Lysis Buffer (Servicebio, China), and transferred into centrifuge tubes followed by centrifugation at 12,000 rpm for 10 min. The supernatant was collected and mixed with 4× sodium dodecyl sulfate, followed by heating at 100 °C for 10 min. Proteins were loaded on 10% sodium dodecyl sulfate-polyacrylamide gel electrophoresis, separated and transferred onto a PVDF membrane. The PVDF membrane was incubated with the corresponding antibody at 4 °C overnight (10 mL of 1 × TBS and Tween-20 (TBST), 5% nonfat milk and antibody), followed by incubation with horseradish peroxidase-conjugated goat anti-rabbit secondary antibody at room temperature for 1 h. After washing in TBST, visualization was performed with an ECL kit (Servicebio, China). The antibodies used for immunoblotting are listed in Table S2.

*OA Induction and Intra-articular Injection of Hydrogels*: The animal investigation received approval from the Ethics Committee of the Third Military Medical University (Approval No.: AMUWEC20230200). Twelve-week-old male Sprague-Dawley (SD) rats were randomly allocated into two groups: sham and OA groups. The OA rats underwent anesthesia via intraperitoneal injection of 2% pentobarbital sodium (0.3 mL/100 g of body weight). Subsequently, the medial meniscus tibial ligament (MMTL) of the knee joint was transected to induce OA. One-week post-surgery, the OA group was further divided into five subgroups, each receiving intra-articular injections of one of the following: PBS, OHA/HA-ADH, MgHCF@OHA/HA-ADH, OHA/HA-ADH/SeMet, or MgHCF@OHA/HA-ADH/SeMet. The injection volume was 100 μL. Each group received a single injection. Except for the sham group, all rats were subjected to treadmill running (XR-PT-10B, Xinruan, Shanghai, China) at a speed of 20 m/min for 30 min/day 3 days a week. At eight weeks post-surgery, the rats were humanely sacrificed, and the collection of knee joints was conducted for further evaluation.

*Histological Evaluation*: The rats were anesthetized, and then, the knees were harvested after heart perfusion, fixed for 24 h in 4% paraformaldehyde, and decalcified for 3 weeks in 10% EDTA (pH = 7.4). The samples were fixed in optimal cutting temperature (OCT) compound (Sakura Finetek) or paraffin. Then, the medial compartment of the samples was longitudinally oriented and cut to 4 μm. Hematoxylin and eosin (H&E) staining was performed to calculate the ratio of the thickness of calcified cartilage (CC) and hyaline cartilage (HC) in cartilage. Three features of chronic synovitis (enlargement of lining cell layer, cellular density of synovial stroma, leukocytic infiltrate) were semiquantitatively evaluated (from 0, absent to 3, strong) and each feature was graded separately. The sum provided the synovitis score, which was interpreted as follows: 0-1, no synovitis; 2-4, low-grade synovitis; 5-9, high grade synovitis (Table S3) [2, 3]. Safranin O-Fast Green (SOFG) staining was performed to examine proteoglycans in cartilage. The tissue samples were mounted after being dehydrated (ethanol). Images were acquired by microscopy (Olympus). Moreover, the sections with SOFG were investigated based on the Osteoarthritis Research Society International (OARSI) standard [4], which could score the materials in four stages (degree of involvement) and six grades (depth of lesion) in the range from 0 (normal) to 24 (severe osteoarthritis). The evaluation of each specimen was conducted was performed in a blinded manner.

*Immunohistochemical and Immunofluorescence Evaluation*: The protein expression in the tissues was assessed by the use of immunochemistry or immunofluorescence techniques. The tissue slices underwent deparaffinization, blocking, and incubation with anti-GPX4 (Afﬁnity, DF6701) and anti-ACSL4 (Afﬁnity, DF12141) antibodies at a dilution ratio of 1:100 or anti-MMP13 (Servicebio, GB11247), anti-COL2 (Servicebio, GB11021), or anti-4-HNE (Bioss, bs-6313R) antibodies at a dilution ratio of 1:200. This process was carried out overnight at a temperature of 4 °C for immunohistochemical staining. The next day, using a DAB peroxidase substrate kit, the slides were exposed to an intensified enzyme-labeled goat anti-rabbit IgG polymer. Immunofluorescent labeling was performed by incubating slices with anti-CD68 (Bioss, bs-20403R, 1:200), anti-CD206 (Proteintech, 18704-1-AP, 1:200), anti-iNOS (Bioss, bs-20601R, 1:200), anti-GPX4 (1:200, Afﬁnity, DF6701) or anti-ACSL4 (1:200, Affinity, DF12141). Subsequently, the sections were treated with secondary antibodies labeled with either fluorescein isothiocyanate (FITC) or tetramethylrhodamine isothiocyanate (TRITC) at a dilution of 1:300, obtained from ZSGB-BIO. The nuclei were subjected to staining with 4′,6-diamidino-2-phenylindole (DAPI) for visualization. The histology picture was obtained employing a Nikon microscope. The acquisition of confocal microscopic images was performed using either a Zeiss laser scanning microscope 710 or a Leica TCS SP8 STED confocal microscope. The antibodies used in this study are shown in Table S2.

*In Vivo Systemic Toxicity Experiments*: After the rats were subjected to euthanasia using a lethal dosage of anesthetics, the main organs (heart, liver, kidney, lung, and spleen) were collected for H&E staining to evaluate pathological changes. Serum was collected after centrifugation, and received blood chemistry examination to analyze the parameters including ALT, AST, BUN, and CREA to evaluate liver and kidney function.

**References**

[1] Meguro R, Asano Y, Iwatsuki H, Shoumura K. Perfusion-Perls and -Turnbull methods supplemented by DAB intensification for nonheme iron histochemistry: demonstration of the superior sensitivity of the methods in the liver, spleen, and stomach of the rat. *Histochem Cell Biol*. 2003;120(1):73-82.

[2] Krenn V, Morawietz L, Burmester G-R, Kinne R W, Mueller-Ladner U, Muller B, Haupl T. Synovitis score: discrimination between chronic low-grade and high-grade synovitis. *Histopathology*. 2006;49(4):358-364.

[3] Zhu J, Yang S, Qi Y, Gong Z, Zhang H, Liang K, Shen P, Huang Y Y, Zhang Z, Ye W, Yue L, Fan S, Shen S, Mikos A G, Wang X, Fang X. Stem cell-homing hydrogel-based miR-29b-5p delivery promotes cartilage regeneration by suppressing senescence in an osteoarthritis rat model. *Sci Adv*. 2022;8(13):eabk0011.

[4] Pritzker K P, Gay S, Jimenez S A, Ostergaard K, Pelletier J P, Revell P A, Salter D, van den Berg W B. Osteoarthritis cartilage histopathology: grading and staging. *Osteoarthritis Cartilage*. 2006;14(1):13-29.


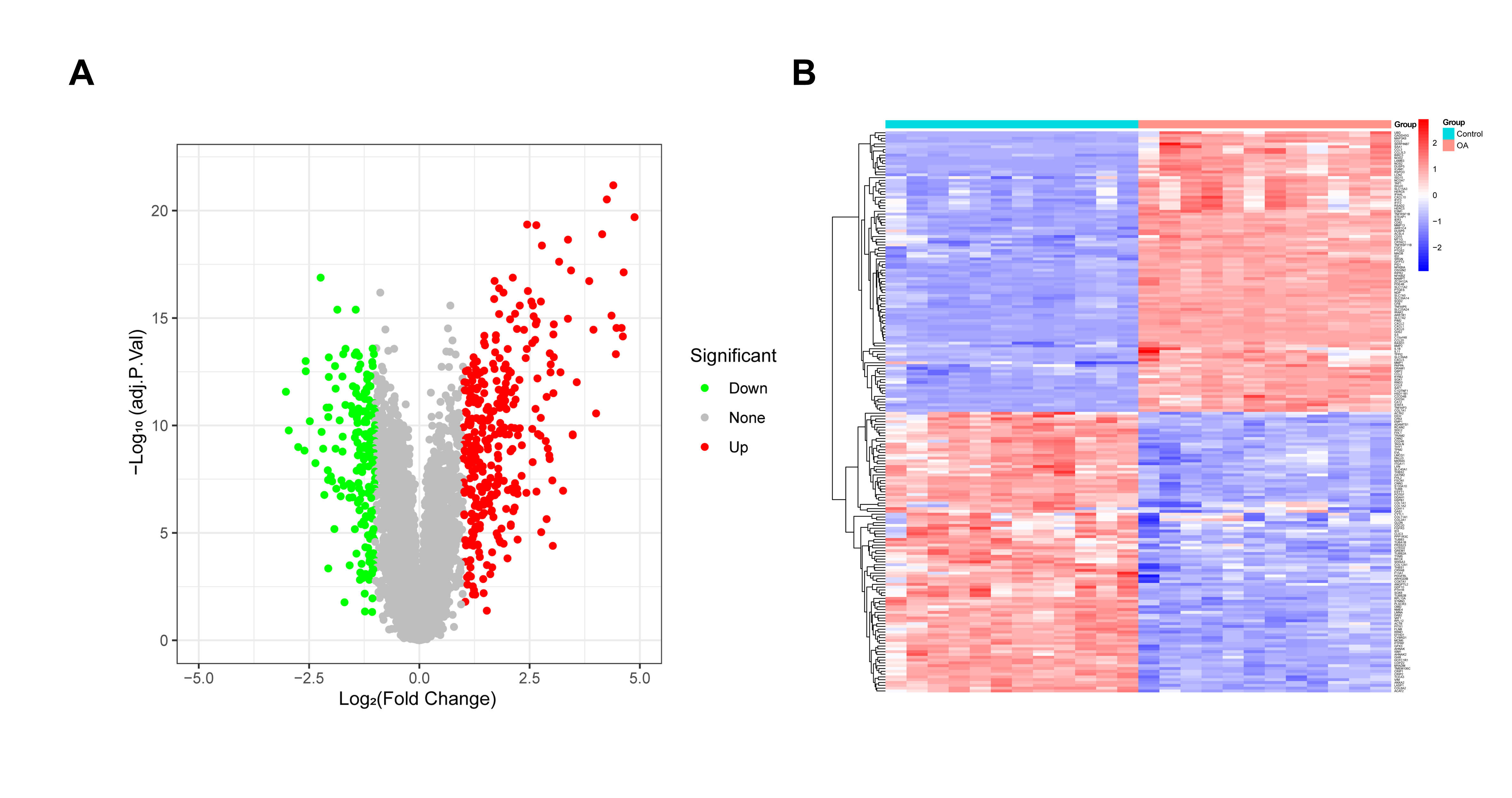


**Fig. S1.**  (A) Volcano plots were generated representing DEGs between the two groups. (B) Heatmap showing that there were differentially expressed genes between the two groups.


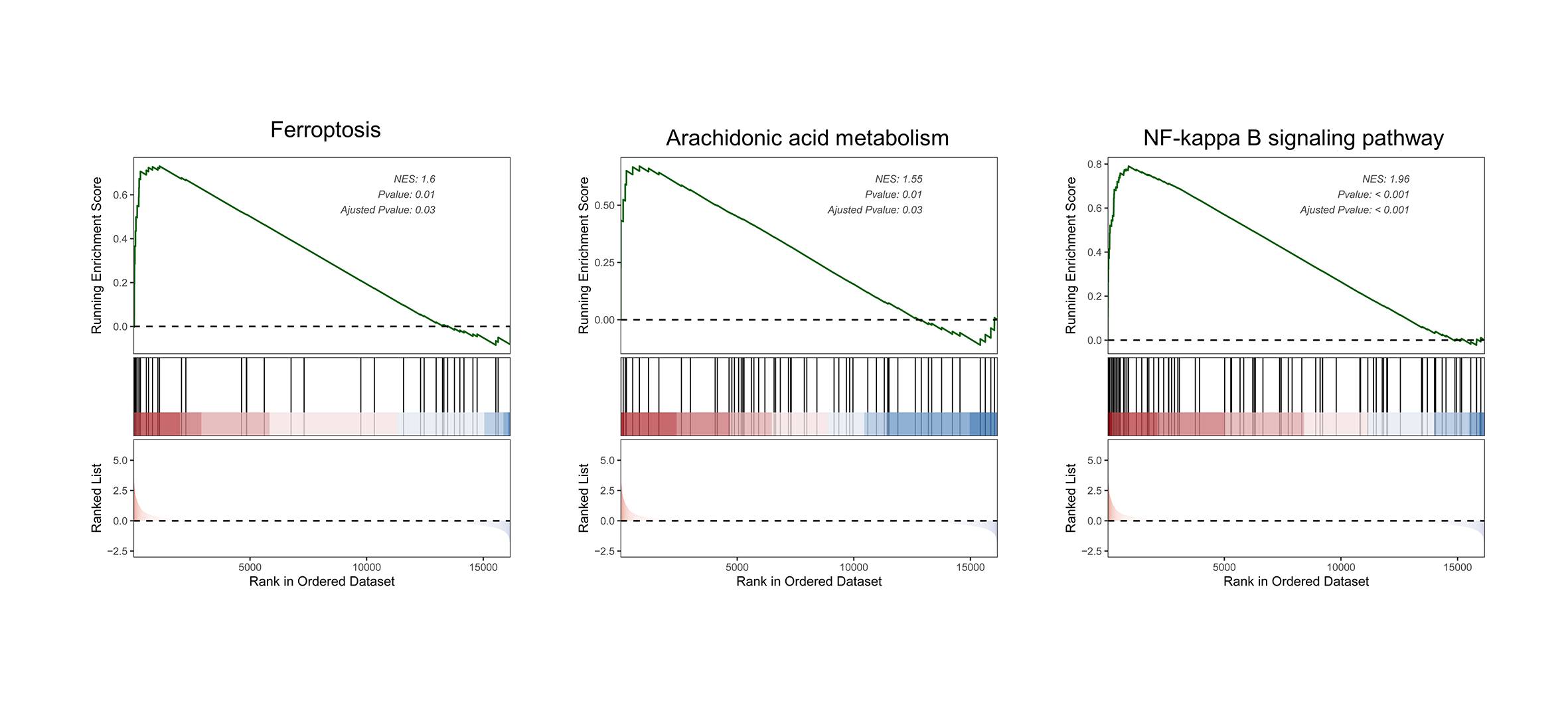


**Fig. S2.** Gene set enrichment analysis (GSEA) was performed to compare the gene sets involved in the ferroptosis, arachidonic acid metabolism, and NF-kappa B signaling pathway between DEGs within the OA group and those within the control group.


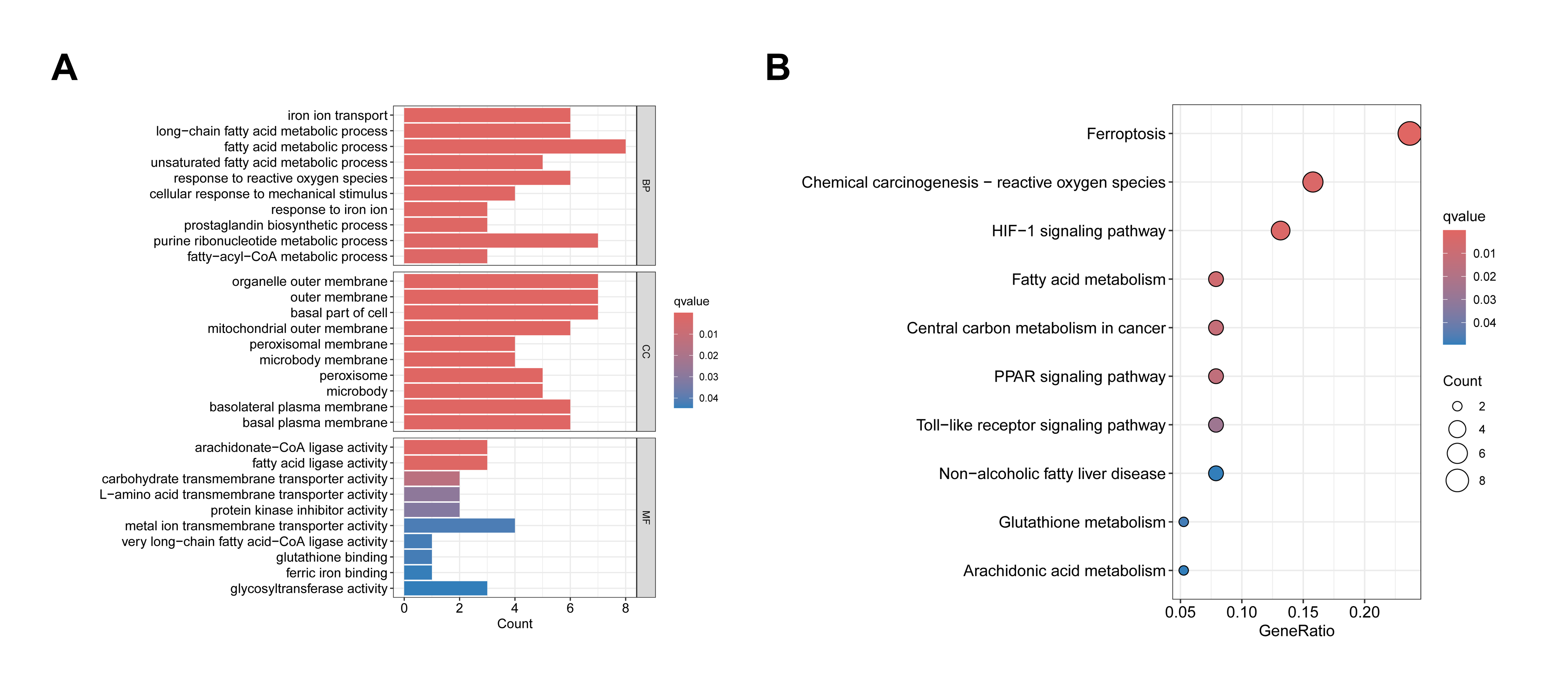


**Fig. S3.** (A) Gene Ontology (GO) enrichment bar plot for FRDEGs based on three sub-ontologies: biological process (BP), cellular component (CC), and molecular function (MF). (B) Kyoto Encyclopedia of Genes and Genomes (KEGG) enrichment analysis of FRDEGs.

**
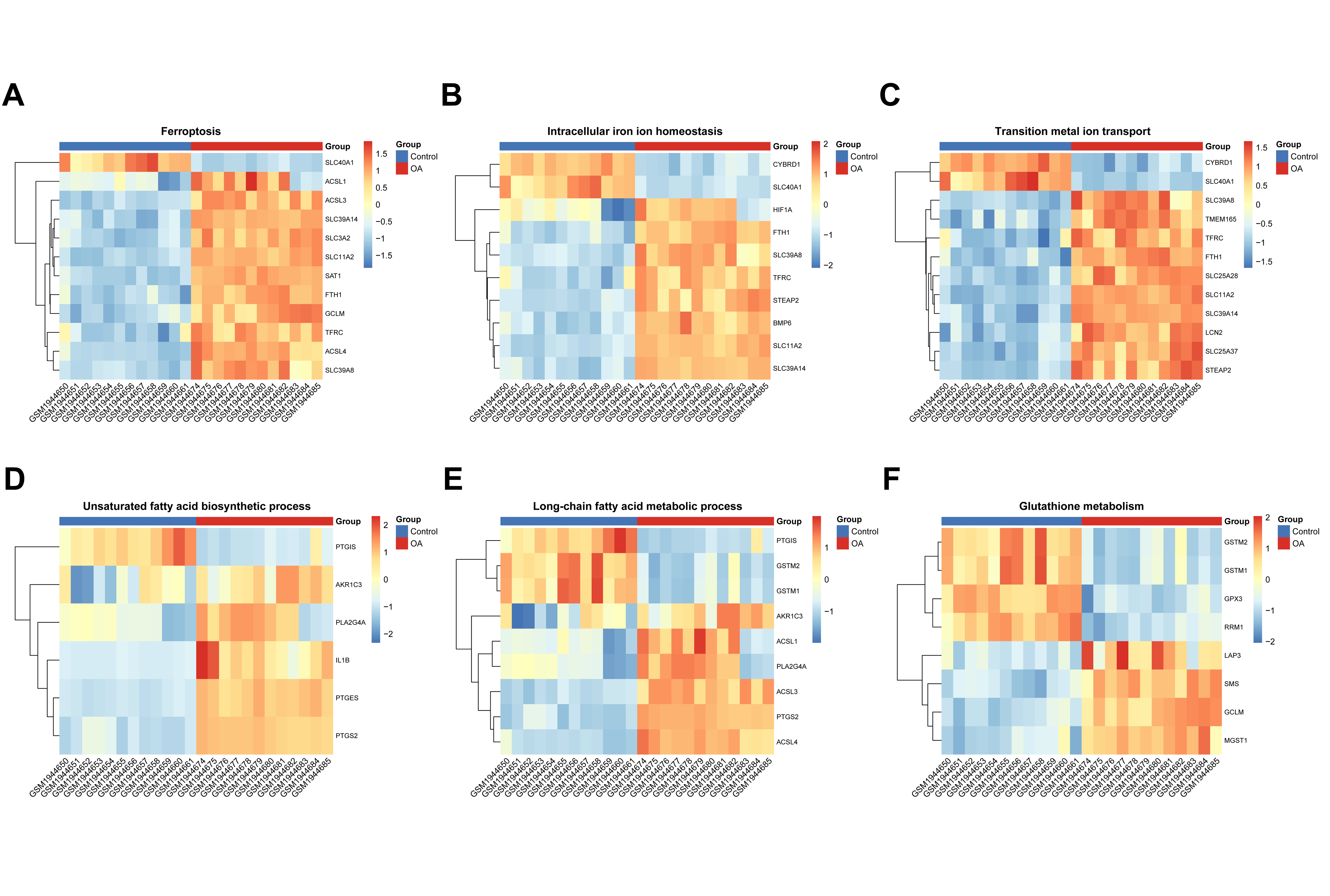
**

**Fig. S4.** (A-F) Heatmap of ferroptosis, intracellular iron ion homeostasis, metal ion transport, unsaturated fatty acid biosynthetic process, long-chain fatty acid metabolic process, and glutathione metabolism related up-regulated and down-regulated gene profile in the OA group compared with the control group.

**
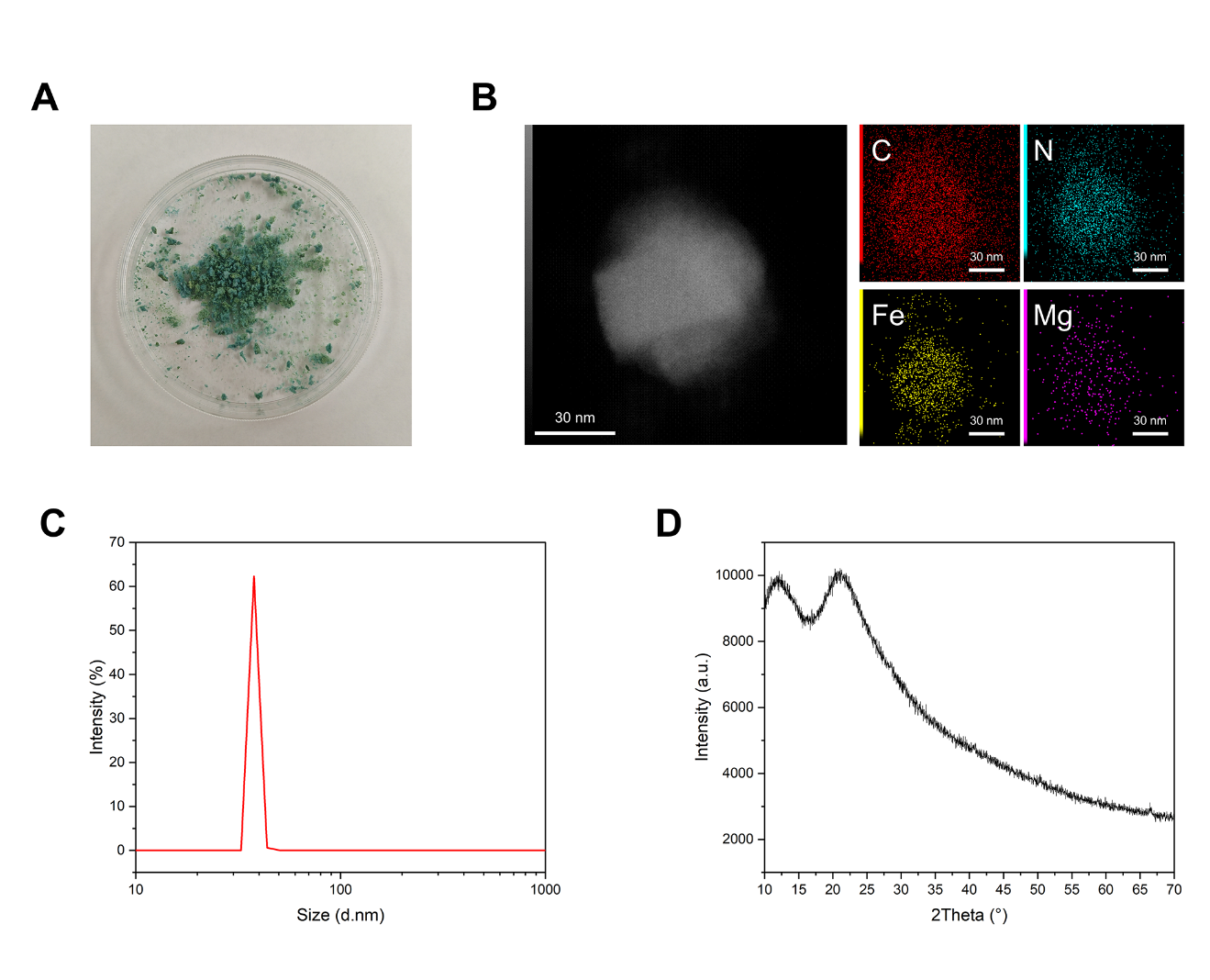
**

**Fig. S5.** (A) MgHCF nanoparticles powders. (B) EDS mapping image of the MgHCF nanoparticles. Scale bar, 30 nm. (C) DLS analysis of MgHCF nanoparticles. (D) XRD pattern spectra of MgHCF nanoparticles.

**
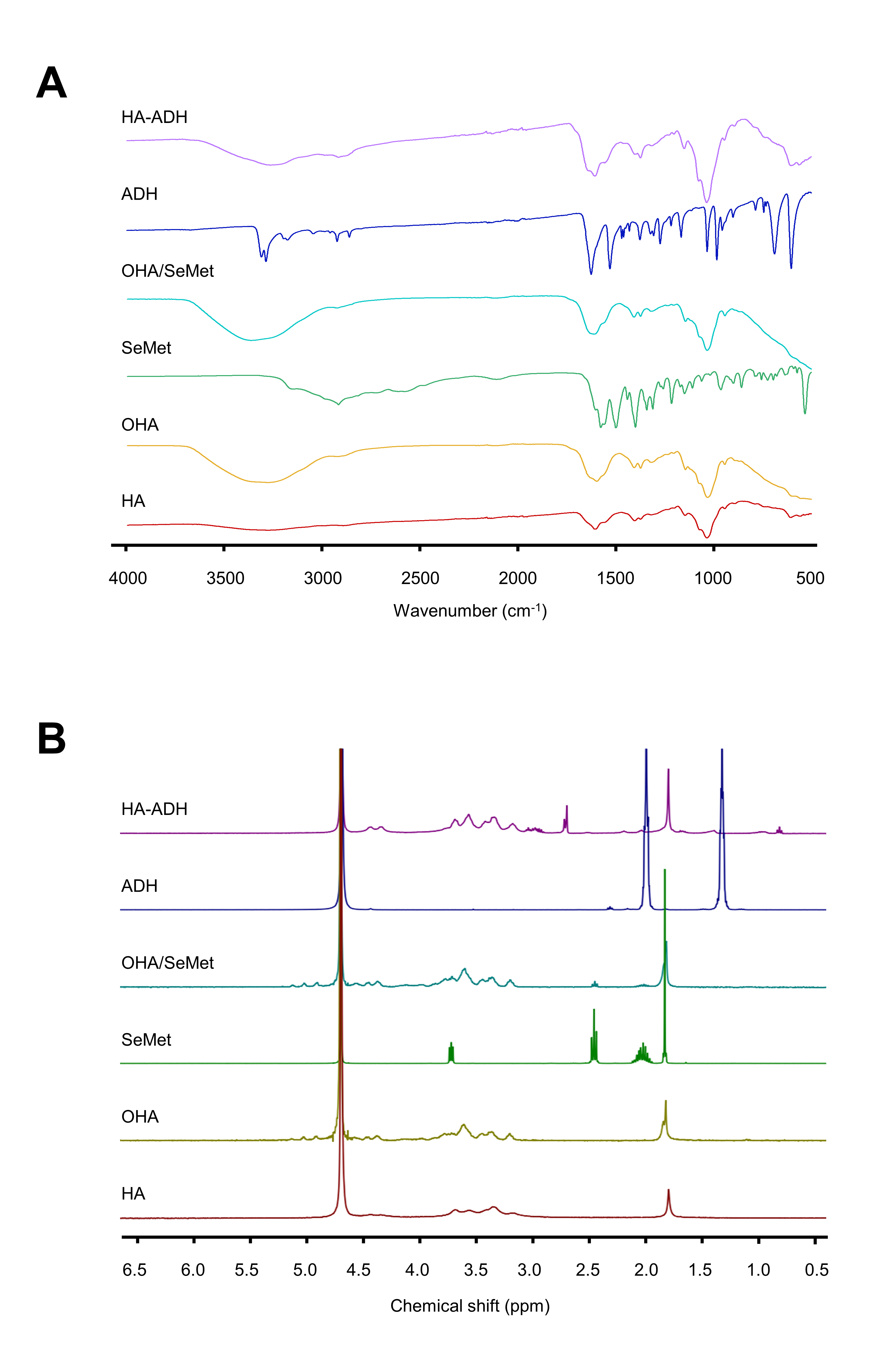
**

**Fig. S6.** (A) FTIR and (B) ^1^H NMR spectrum of HA, OHA, SeMet, OHA/SeMet, ADH, and HA-ADH.

**
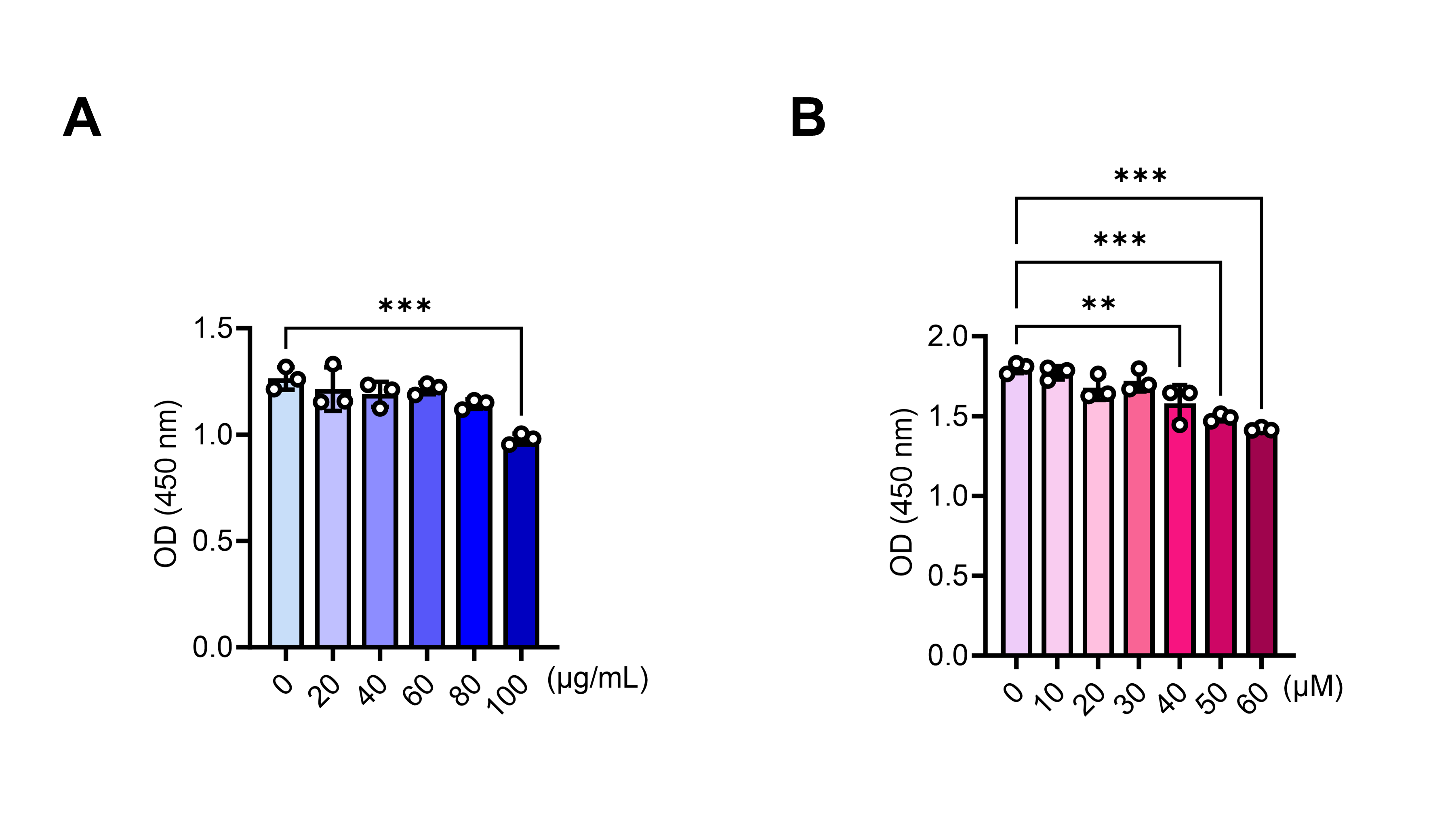
**

**Fig. S7.** Cell viability of C28/I2 cells treated with different concentrations of (A) MgHCF or (B) SeMet. Data were represented as mean ± SD. NS meant not significant, **p* < 0.05, ***p* < 0.01, and ****p* < 0.001.

**
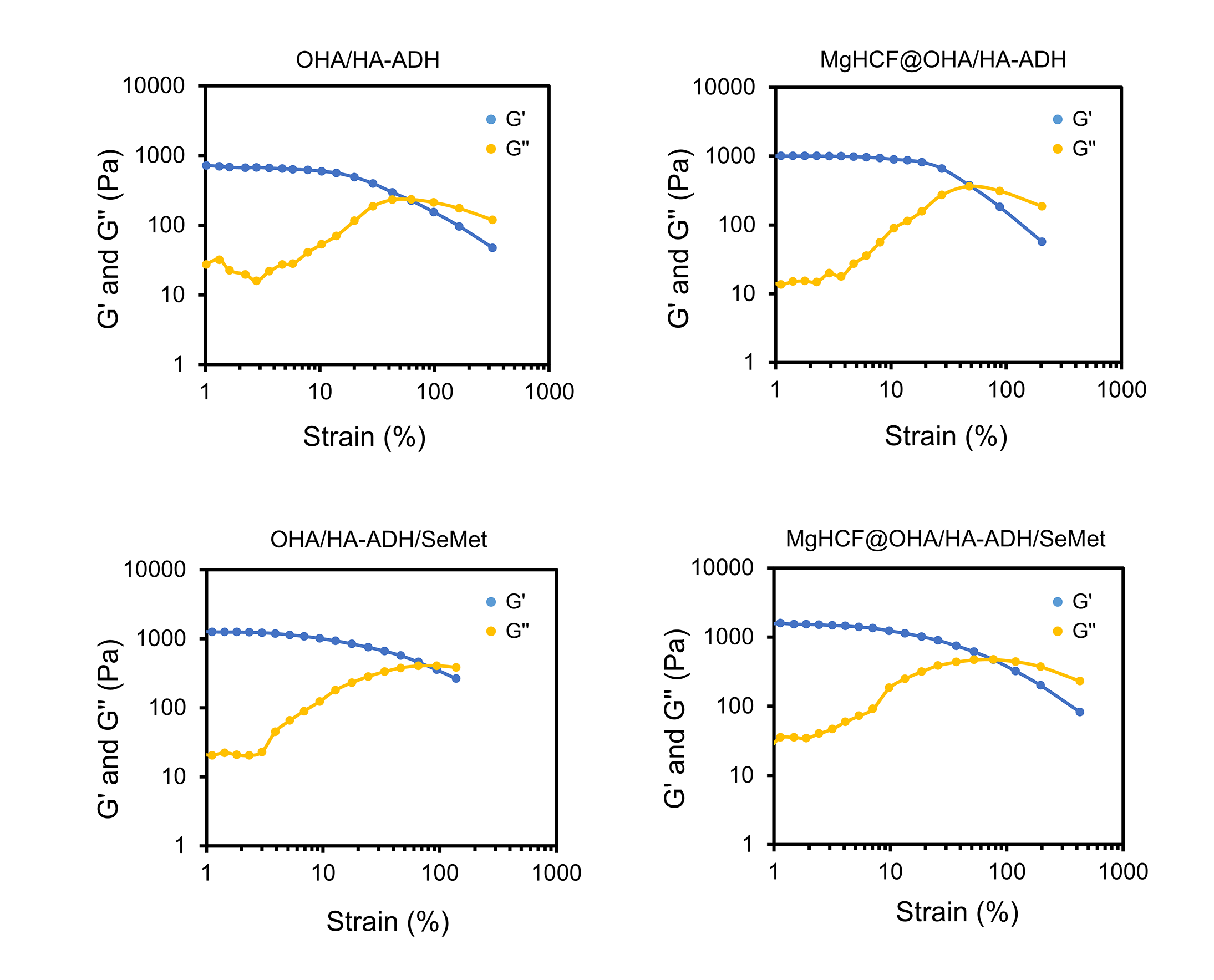
**

**Fig. S8.** Strain sweep of the hydrogel samples with the strain ranging from 1% to 1000% at a frequency of 1 Hz.


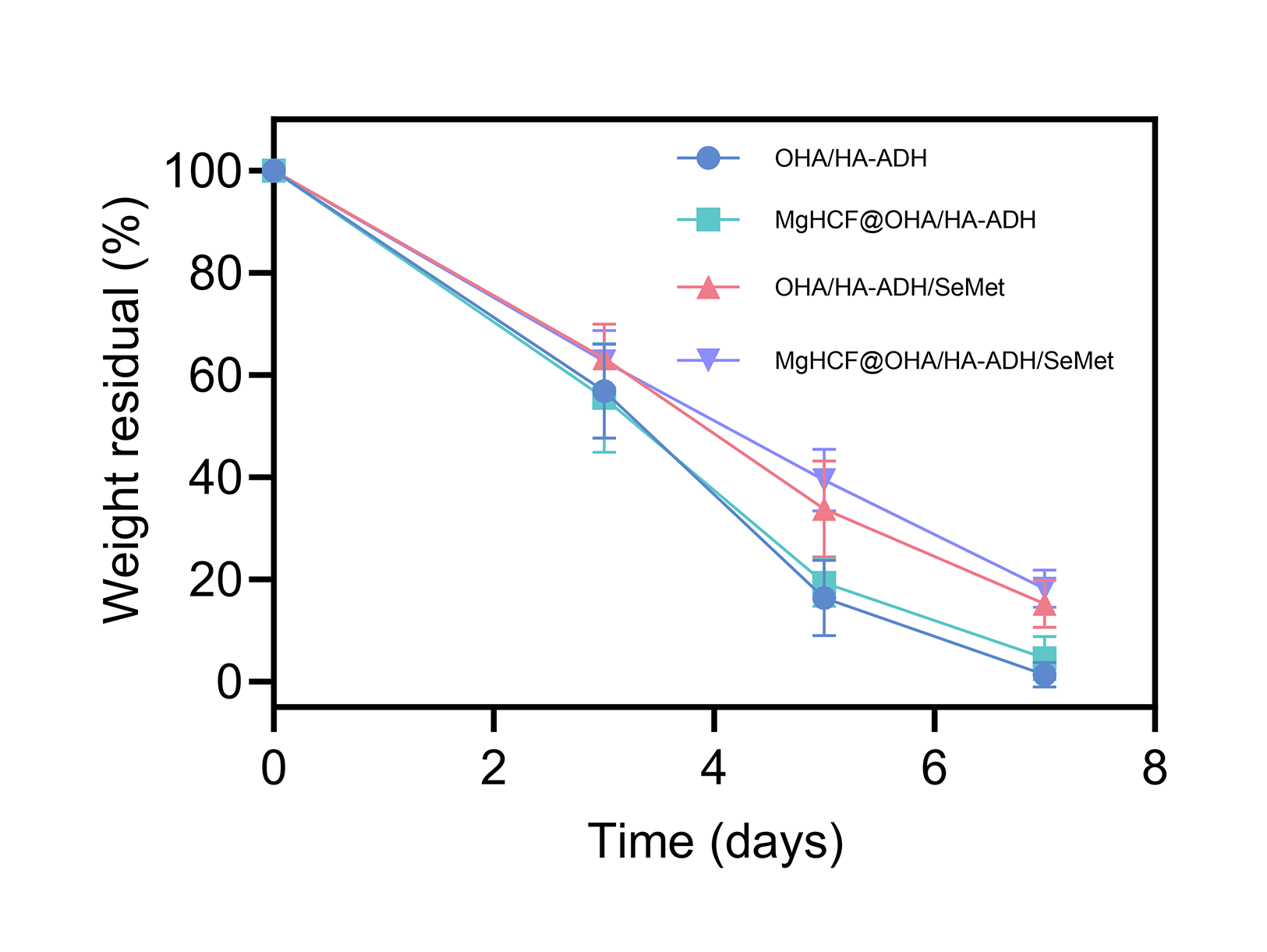


**Fig. S9.** Degradation curves of the four groups of hydrogels in PBS.


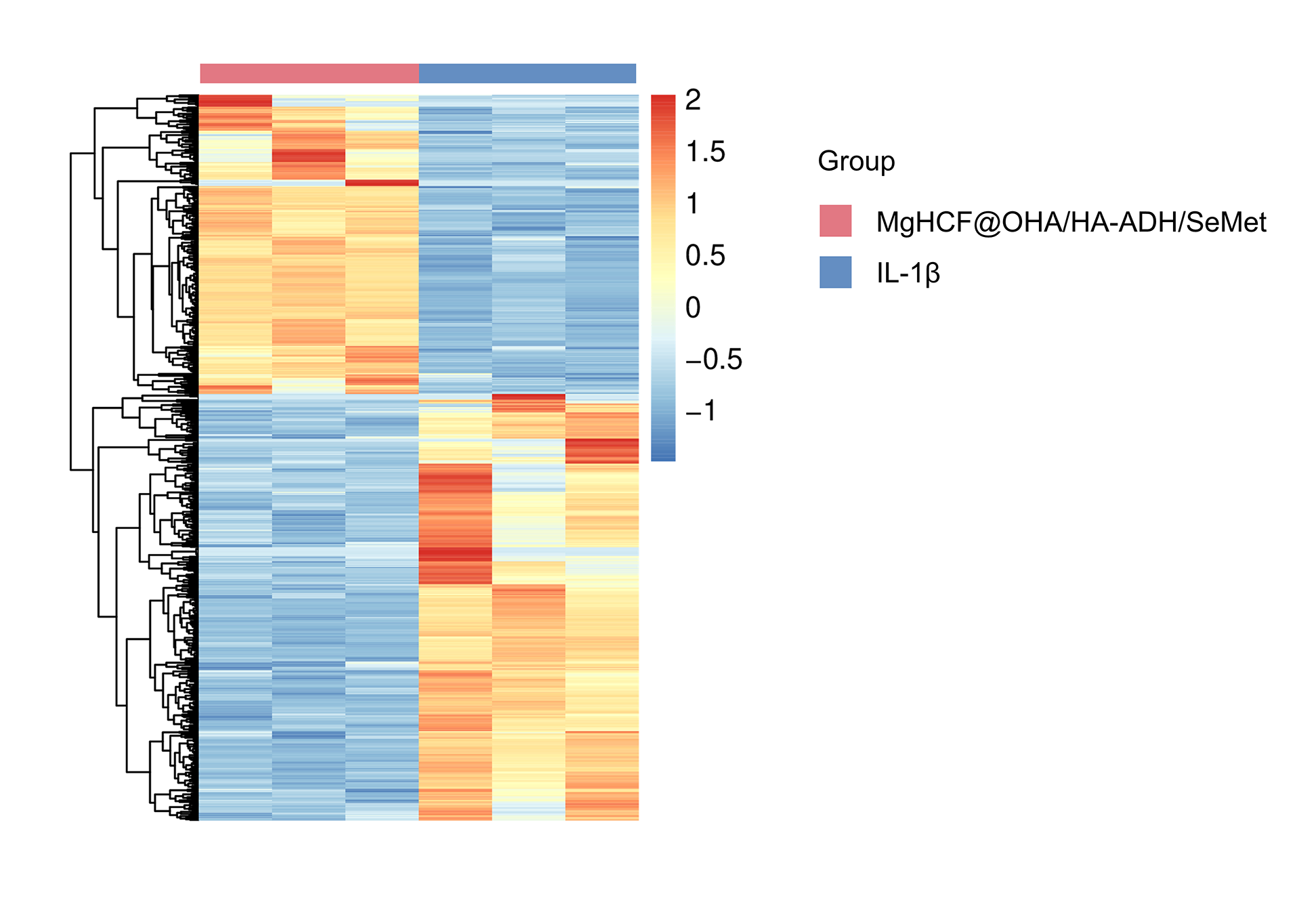


**Fig. S10.** Heatmap showing that there were DEGs between the IL-1β plus MgHCF@OHA/HA-ADH/SeMet group and the IL-1β group.


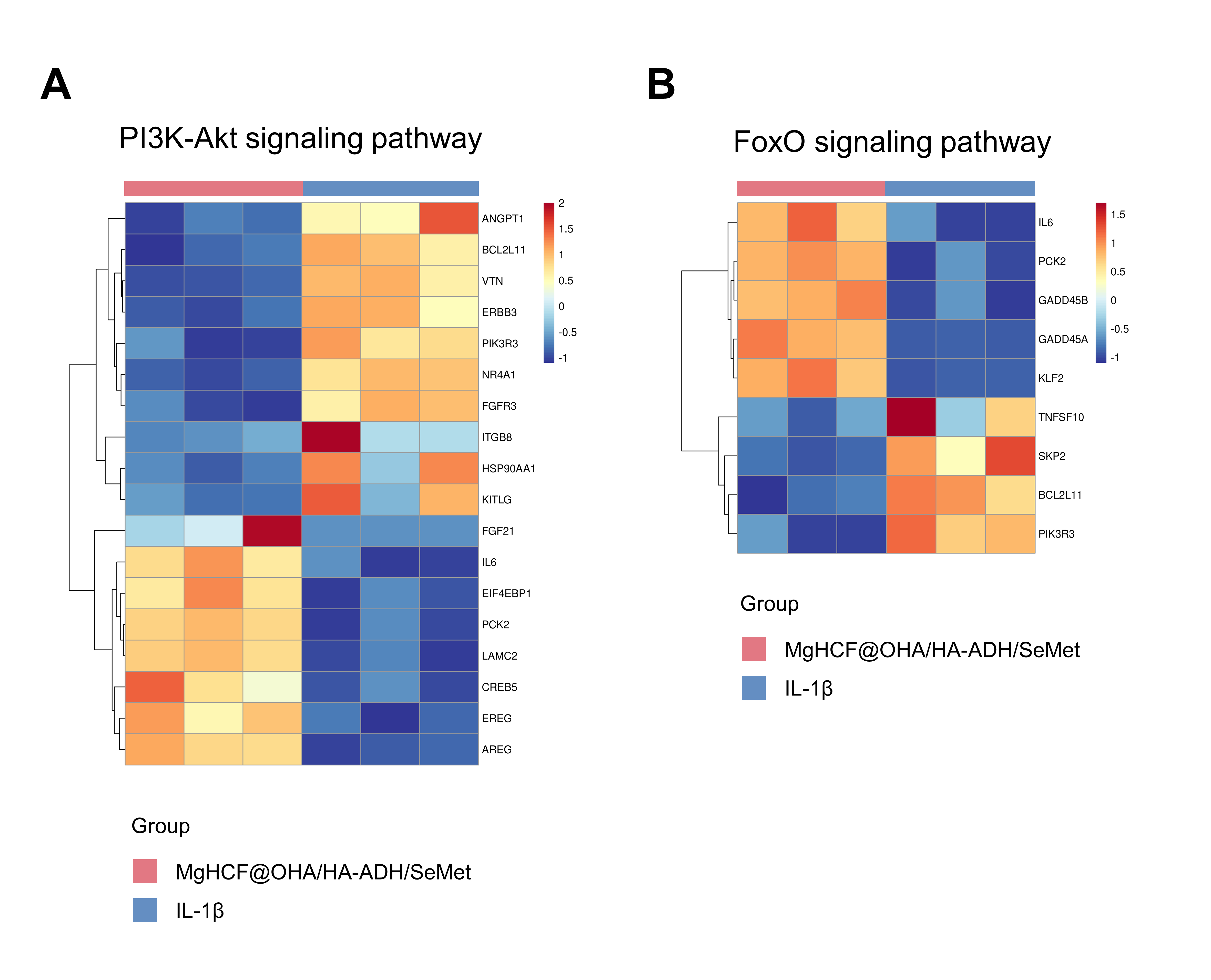


**Fig. S11.** (A) Heatmap of differentially expressed genes associated with enrichment PI3K-Akt signaling pathway for IL-1β plus MgHCF@OHA/HA-ADH/SeMet group compared to IL-1β group. (B) Heatmap of differentially expressed genes associated with enrichment FoxO signaling pathway for IL-1β plus MgHCF@OHA/HA-ADH/SeMet group compared to IL-1β group.


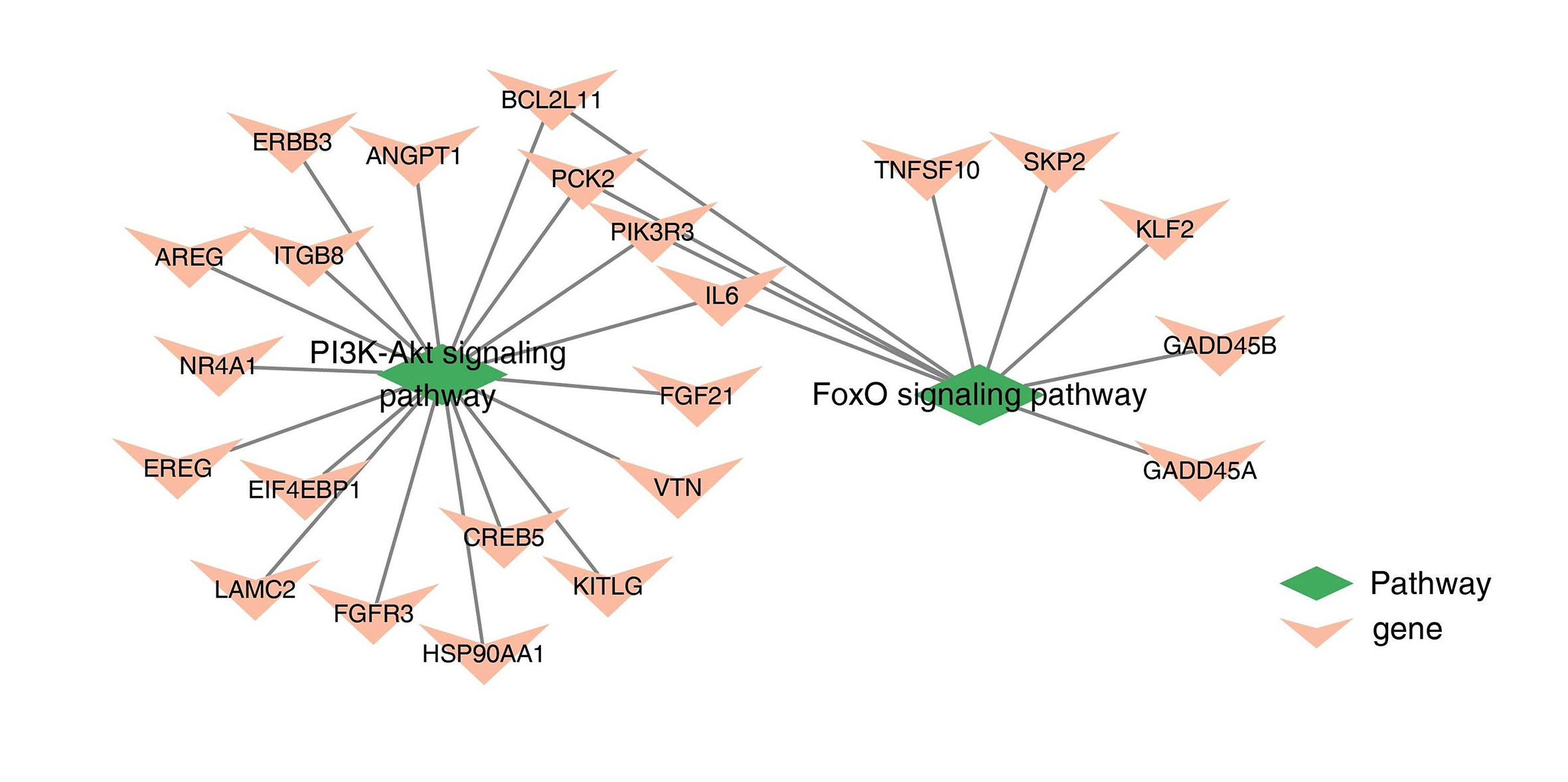


**Fig. S12.** Cluster analysis of PI3K-Akt signaling pathway and FoxO signaling pathway.


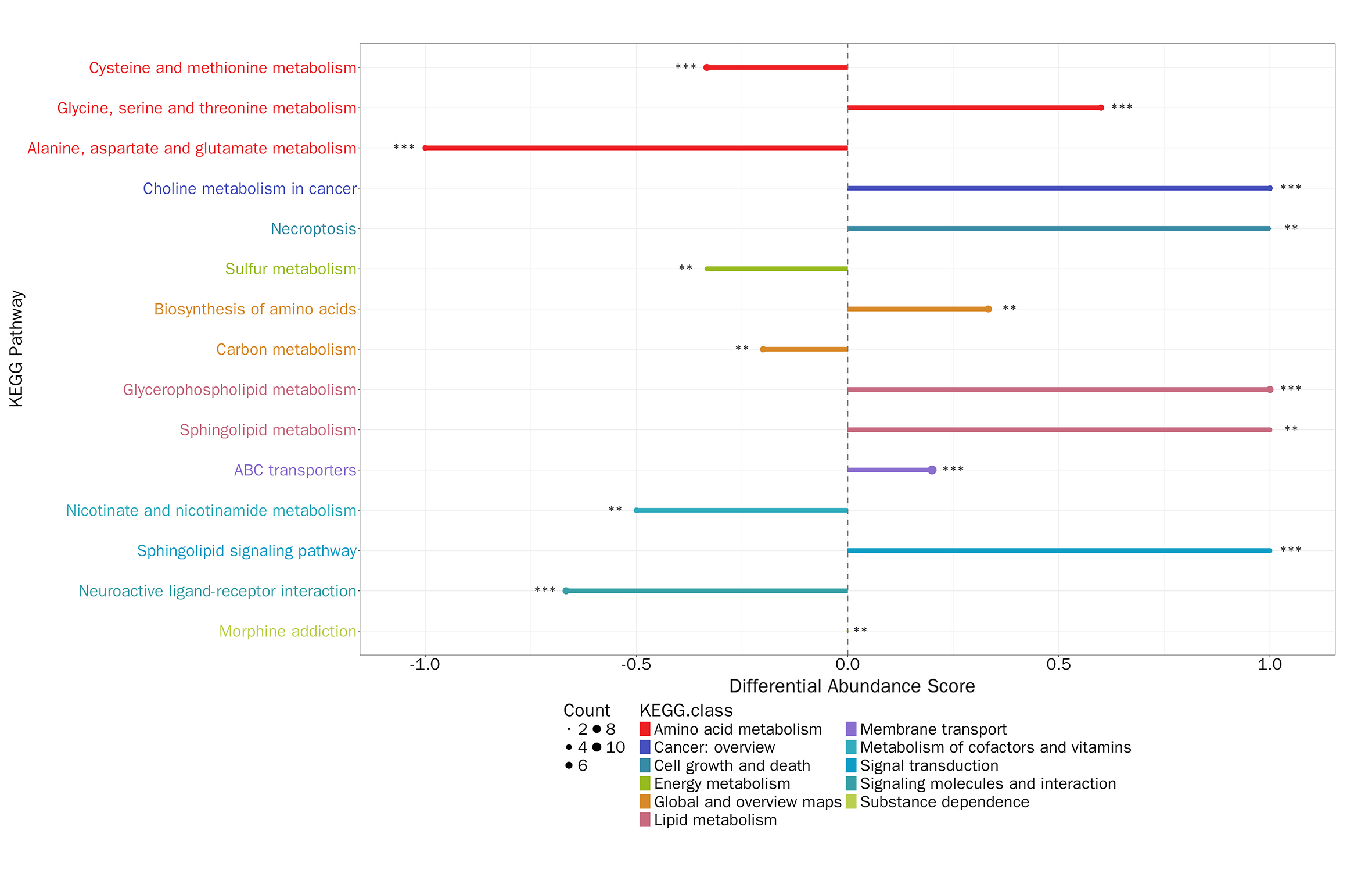


**Fig. S13.** KEGG enrichment analysis of the pathways involved in the biological effect induced by IL-1β or IL-1β plus MgHCF@OHA/HA-ADH/SeMet treatment based on the metabolomics study.


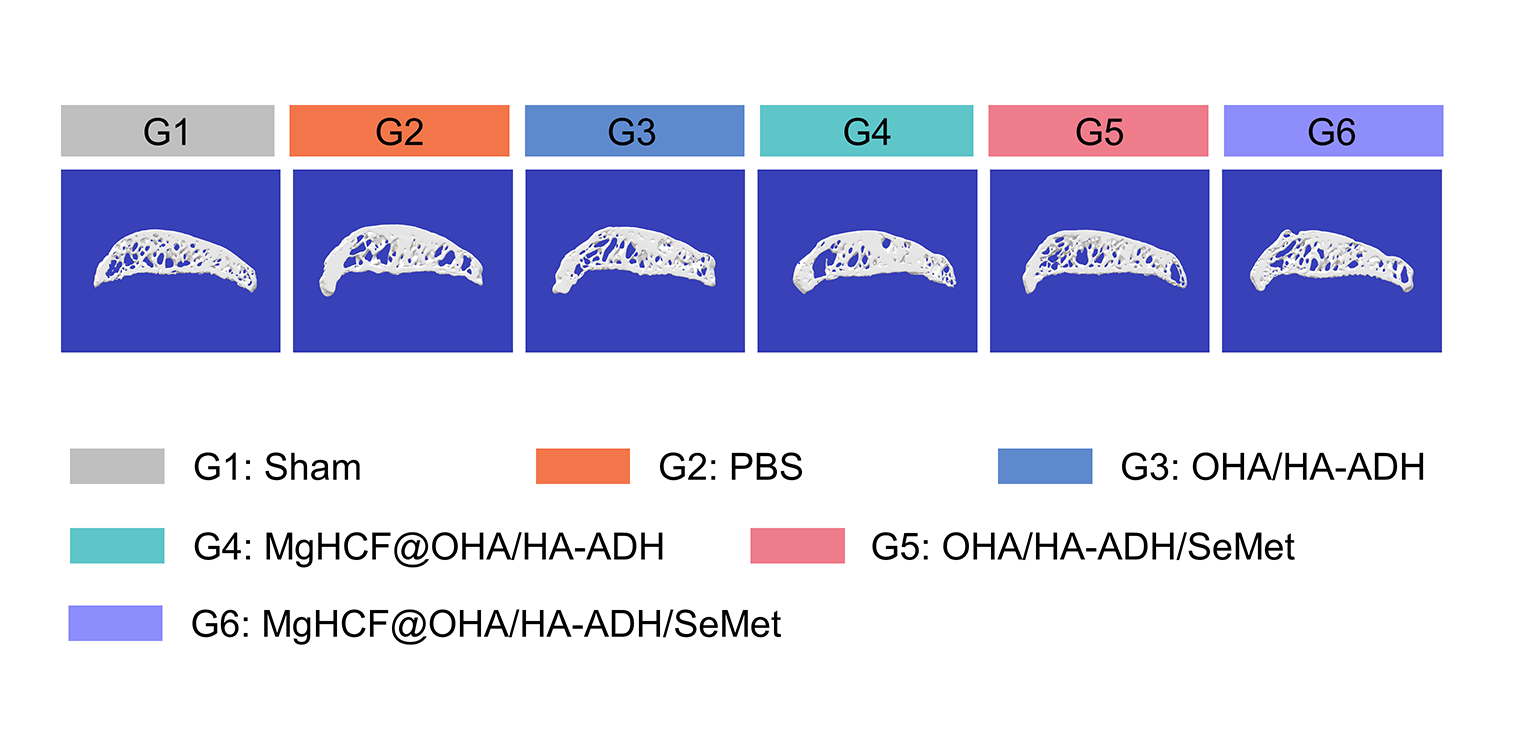


**Fig. S14.** Micro-CT reconstruction of medial subchondral bone.


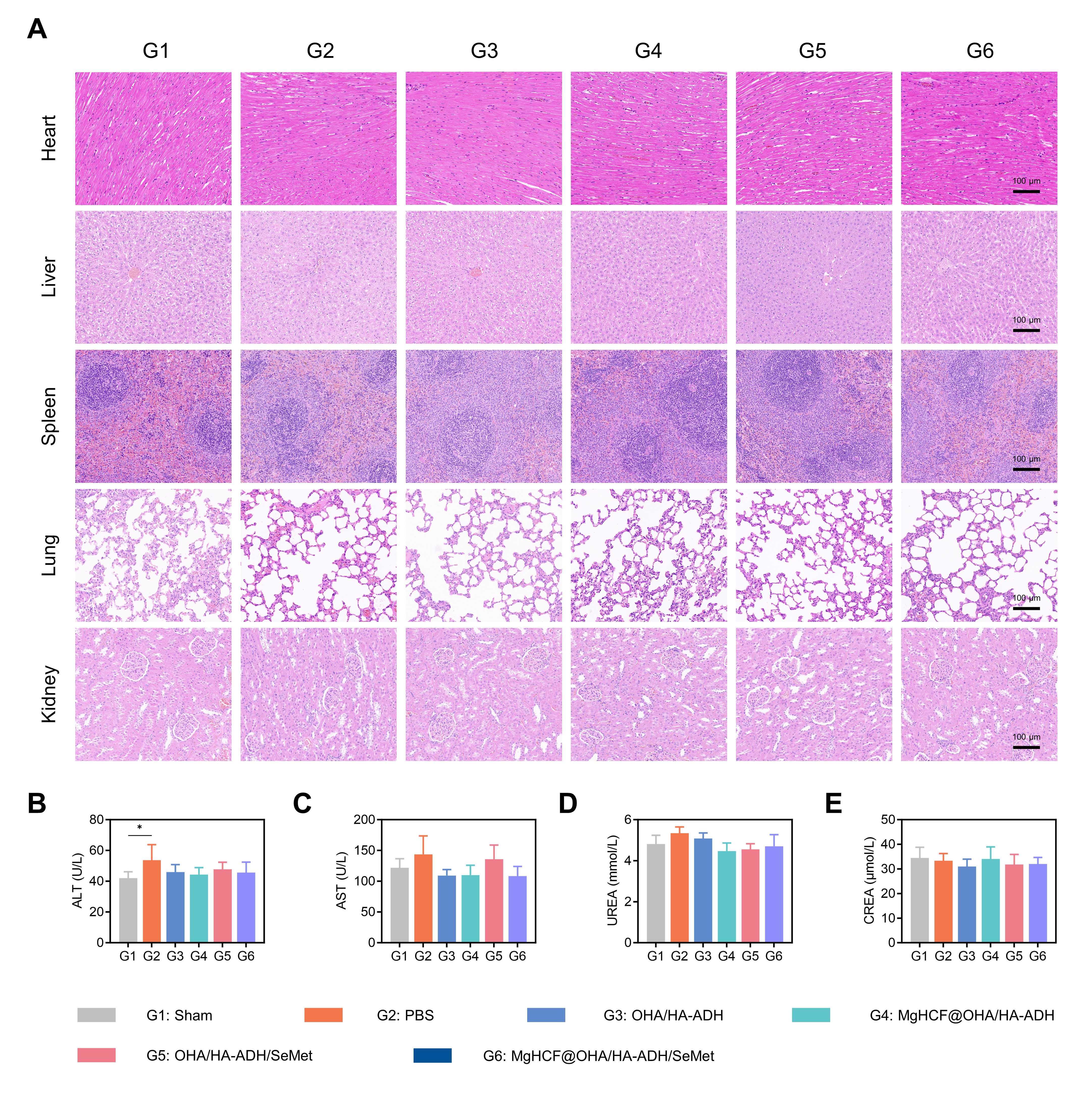


**Fig. S15.** (A) *In vivo* biocompatibility analysis by H&E staining of major organ sections of rats. Scale bar, 100 µm. (B) ALT, (C) AST, (D) UREA, and (E) CREA level in serum of rats treated with the hydrogels eight weeks after surgery. n = 4-6. Data were represented as mean ± SD. NS meant not significant, **p* < 0.05, ***p* < 0.01, and ****p* < 0.001.

**Table S1. Sequences of primers used for the real-time quantitative PCR analysis.**

| **Gene** | **Species** | **Direction** | **Sequences 5’-3’** |
| --- | --- | --- | --- |
| SLC40A1 | Human | Forward | GTGGATCCTTGGCCGACTAC |
|  |  | Reverse | AAGTGCCACATCCGATCTCC |
| NOX1 | Human | Forward | TTGGGTCAACATTGG CCTGT |
|  |  | Reverse | AAGGACAGCAGATTGCGACA |
| SLC7A11 | Human | Forward | TGGTCAGAAAGCCTGTTGTGT |
|  |  | Reverse | CTCCTTGTTGCCCAGGGAAG |
| GPX4 | Human | Forward | AGAGATCAAAGAGTTCGCCGC |
|  |  | Reverse | TCTTCATCCACTTCCACAGCG |
| ACSL4 | Human | Forward | GGAATGACAGGCCAGTGTGA |
|  |  | Reverse | TAGCACATGAGCCAAAGGCA |
| TFRC | Human | Forward | CTGGCTCGGCAAGTAGATGG |
|  |  | Reverse | TGTGACATTGGCCTTTGTGT |
| PTGS2 | Human | Forward | GGCCATGGGGTGGACTTAAA |
|  |  | Reverse | TGAAAAGGCGCAGTTTACGC |
| β-actin | Human | Forward | GCGGGAATCCTGAAGGAGAATGC |
|  |  | Reverse | TCAAGTTTGCCAGTCACCTCTAAGC |

**Table S2. Antibody information used in this study.**

| **Antibody** | **Company** | **Catalog #** | **Application/Dilution** |
| --- | --- | --- | --- |
| GPX4 | Afﬁnity | DF6701 | IHC 1:100, IF 1:200 |
| ACSL4 | Afﬁnity | DF12141 | IHC 1:100, IF 1:200 |
| 4-HNE | Bioss | bs-6313R | IHC 1:200 |
| MMP13 | Servicebio | GB11247 | IHC 1:200 |
| MMP13 | Bioworld | BS79990 | IF 1:200 |
| COL2 | Servicebio | GB11021 | IHC 1:200 |
| COL2 | Bioss | bs-10589R | IF 1:200 |
| p-PI3K | Bioss | bs-5570R | WB 1:1000 |
| PI3K | Bioworld | AP0230 | WB 1:1000 |
| p-Akt | CST | 4060T | WB 1:2000 |
| Akt | Bioworld | AP0489 | WB 1:1000 |
| p-FoxO1 | CST | 9464T | WB 1:1000 |
| FoxO1 | CST | 2880T | WB 1:1000 |
| FoxO1 | Bioss | bs-2537R | IF 1:200 |
| CD206 | Proteintech | 18704-1-AP | IF 1:200 |
| iNOS | Bioss | bs-20601R | IF 1:200 |
| CD68 | Bioss | bs-20403R | IF 1:200 |
| β-actin | ABclonal | AC038 | WB 1:10000 |
| GAPDH | ABclonal | A19056 | WB 1:50000 |

**Table S3. Scheme for the histopathological assessment of the three features of chronic synovitis.**

| Enlargement of the synovial lining cell layer | |
| --- | --- |
| 0 | The lining cells form one layer |
| 1 | The lining cells form 2-3 layers |
| 2 | The lining cells form 4-5 layers, few multinucleated cells might occur |
| 3 | The lining cells form more than 5 layers, the lining might be ulcerated and multinucleated cells might occur |
| Density of the resident cells | |
| 0 | The synovial stroma shows normal cellularity |
| 1 | The cellularity is slightly increased |
| 2 | The cellularity is moderately increased, multinucleated cells might occur |
| 3 | The cellularity is greatly increased, multinucleated giant cells, pannus formation and rheumatoid granulomas might occur |
| Inflammatory infiltrate | |
| 0 | No inflammatory infiltrate |
| 1 | Few mostly perivascular situated lymphocytes or plasma cells |
| 2 | Numerous lymphocytes or plasma cells, sometimes forming follicle-like aggregates |
| 3 | Dense band-like inflammatory infiltrate or numerous large follicle-like aggregates |
| Sum 0 or 1 | No synovitis |
| Sum 2-4 | Low-grade synovitis |
| Sum 5-9 | High-grade synovitis |
